# Water Regime Determines Dose-Dependent Growth and Biomass Allocation Responses of Soybean Seedlings to *Bacillus subtilis* and *Bacillus amyloliquefaciens*

**DOI:** 10.3390/plants15152267

**Published:** 2026-07-24

**Authors:** João Paulo Alves da Silva, Luis Guilherme Teixeira Crusiol, José Salvador Simoneti Foloni, José Renato Bouças Farias, Marcelo Luiz Chicati, Roney Berti de Oliveira, Renato Herrig Furlanetto, José A. M. Demattê, Marcos Rafael Nanni, Renan Falcioni

**Affiliations:** 1 Graduate Program in Agronomy, State University of Maringá, Av. Colombo, 5790, Maringá 87020-900, PR, Brazil; ra125202@uem.br (J.P.A.d.S.); mlchicati@uem.br (M.L.C.); rboliveira@uem.br (R.B.d.O.); mrnanni@uem.br (M.R.N.); 2 National Soybean Research Center, Brazilian Agricultural Research Corporation, Rodovia Carlos João Strass, s/n°, Distrito de Warta, Londrina 86001-970, PR, Brazil; luis.crusiol@colaborador.embrapa.br (L.G.T.C.); salvador.foloni@embrapa.br (J.S.S.F.); joserenato.farias@embrapa.br (J.R.B.F.); 3 Gulf Coast Research and Education Center, University of Florida, Wimauma, FL 33598, USA; re.herrigfurlane@ufl.edu; 4 Department of Soil Science, Luiz de Queiroz College of Agriculture, University of São Paulo, Av. Pádua Dias, 11, Piracicaba 13418-260, SP, Brazil; jamdemat@usp.br

**Keywords:** biomass partitioning, drought acclimation, leaf construction, microbial biostimulation, rhizosphere preconditioning, seedling establishment, thermal phenotyping

## Abstract

Water limitation during soybean (*Glycine max* (L.) Merr.) establishment restricts leaf expansion and root–shoot development, whereas microbial biostimulants may modify these responses only within particular moisture contexts. We tested whether a mixed-species suspension of *Bacillus subtilis* and *Bacillus amyloliquefaciens* produced water-regime-dependent dose responses in a controlled-environment tray-cell experiment. The seedlings received a single rhizosphere-directed application of 0, 0.05, 0.1, 0.2, 0.4, 0.6, 0.8, 1.0, 1.2, 1.75 or 2.0 mL of undiluted inoculum per cell before exposure for 15 days to full irrigation (W100), 50% or 25% of the W100 replacement volume (W50 and W25), or no irrigation (W0). Each of the 44 treatment combinations comprised six independent biological replicates (264 cells). Two-way ANOVA detected significant water-regime, dose and interaction effects for most thermal, growth, biomass and allocation traits, with water regime as the dominant source of variation. Averaged across doses, relative to W100, W0 increased the leaf-surface temperature by 8.8% and reduced the root length by 44.3%, shoot length by 25.4%, stem diameter by 38.2%, leaf area by 72.3%, total dry mass by 20.1% and specific leaf area by 70.4% on average. The concurrent 17.3% increase in the leaf mass fraction indicated the retention of proportional leaf dry-matter investment despite strongly restricted surface expansion. The bacterial response was non-linear and water-regime-specific. Within W0, 0.05 mL increased root dry mass by 116.7% and total dry mass by 39.3% relative to the untreated W0 control. The highest integrated mean z-score occurred at 0.05 mL under W0, 0.2 mL under W25, 1.2 mL under W50 and 0.2 mL under W100. Correlation analysis identified coordinated trait covariation, whereas hierarchical clustering and principal component analysis separated a warm, low-expansion W0 phenotype from a cool, high-vigour W100 phenotype. These findings provide a quantitative experimental basis for selecting candidate pre-drought doses for seedling-stage screening. Validation across soils, application timings, formulation persistence and reproductive-stage yield are required before commercial recommendation.

## 1. Introduction

Soybean (*Glycine max* (L.) Merr.) establishment depends on rapid hydraulic continuity between the substrate, roots and expanding shoots [1,2]. When the water supply falls below evaporative demand, reduced turgor, stomatal regulation, stress signalling and altered carbon allocation constrain cell expansion before all biomass pools are depleted [1,2,3,4,5,6]. In soybean, progressive water limitation results in coordinated thermal, architectural, metabolic and photosynthetic changes [7], while the magnitude of the field response varies among genotypes and production environments [8]. This early phase is agronomically important because restricted leaf deployment and root exploration can amplify short-term fluctuations in substrate water availability and delay canopy closure [7,8].

Management strategies for water-limited establishment increasingly include manipulation of the rhizosphere microbiome [9,10]. Plant-associated microorganisms can influence nutrient mobilisation, root development, phytohormone balance, osmotic adjustment, redox homeostasis and post-stress recovery [9,10,11]. These effects are conditional rather than universal. Drought-driven microbiome restructuring [12], microbial maintenance of root growth [13], microbiota–endodermis control of mineral homeostasis [14], host preference [15] and stress-specific growth promotion [16] all indicate that outcomes depend on plant genotype, microbial identity and environmental history. Recent field and controlled studies further show that microbial responses vary with crop stage, water supply and microbial combination [17,18,19]. Microbial products should therefore be evaluated as context-dependent modifiers of plant performance rather than as substitutes for irrigation or as inputs expected to produce the same response across moisture environments.

Plant growth-promoting rhizobacteria comprise spore-forming and non-spore-forming taxa, including *Bacillus*, *Pseudomonas* and *Azospirillum* [20,21,22,23]. Compared with many non-spore-forming inoculants, Bacillus has an operational advantage because resistant endospores can improve survival during manufacture, storage and environmental exposure [21,22], whereas rhizosphere-competent strains may influence nutrient acquisition, root growth and stress signalling [20,21,22,23,24]. Additional plant-growth-promoting mechanisms may include 1-aminocyclopropane-1-carboxylate (ACC) deaminase-mediated modulation of stress ethylene under abiotic stress [20,21,23,25], and studies in soybean confirm that plant-associated bacteria can modify drought responses under defined experimental conditions [26]. Nevertheless, positive responses depend on host–microbe compatibility, viable population dynamics, formulation and water availability [10,15,21,24,26]. These limitations are consistent with the broader biostimulant, microbial-consortium and microbiome-engineering literature [27,28,29]. Thus, endospore formation supports product stability, but it does not establish universal biological superiority.

Growth and biomass-allocation analyses provide a mechanistically informative bridge between drought physiology and microbial biostimulation. Root architecture and root elongation are closely linked to hydraulic acclimation and plasticity [30,31,32,33], whereas shoot elongation, leaf expansion, organ dry mass, mass fractions and specific leaf area (SLA) provide complementary phenotyping information on growth and carbon investment [34,35,36,37]. In particular, a lower SLA accompanied by maintenance or an increase in the leaf mass fraction (LMF) indicates that less leaf surface is deployed per unit of leaf biomass. This conservative construction pattern may reduce the exposed transpiring area while retaining a substantial fraction of whole-plant biomass in leaves [36,37]. Examining absolute growth together with allocation ratios can therefore reveal microbial effects that would be obscured by plant size alone [34,35,36,37]. Thermal imaging can complement these measurements by providing a non-destructive, integrative indicator of plant stress, provided that radiometric temperature is interpreted alongside plant architecture and environmental conditions [38,39]. Combining thermal and growth phenotyping is consistent with broader priorities for drought-smart soybean improvement [40].

Recent work has begun to resolve microbial effects across crop stages or contrasting water levels. Arbuscular mycorrhizal (AM) fungal–plant growth-promoting rhizobacteria (PGPR) co-inoculation produced stage-dependent responses in dryland maize [17], whereas controlled studies using *B. amyloliquefaciens* in wheat and *B. subtilis* in maize reported water-level-dependent changes under three water supplies [18,19]. These studies support environmental conditionality but use different crops, microbial consortia and fixed inoculation treatments [17,18,19]. The controlled studies cited above evaluated defined microbial treatments at fixed inoculation levels [17,18,19,26], whereas the broader reviews address mechanisms, formulation, microbial consortia and microbiome engineering without resolving broad dose series across several water regimes [21,23,27,28,29]. Such designs cannot determine whether the effective dose changes along a water-supply gradient, whether the response is monotonic, or whether a dose that is favourable under moderate limitation remains favourable when water is nearly absent. Accordingly, factorial soybean assays that combine a broad inoculum-dose series with multiple operational water regimes, while retaining individual tray cells as the experimental unit, remain insufficiently represented in the literature considered here [17,18,19,21,26,27,28,29].

We therefore evaluated eleven doses of a mixed *B. subtilis* + *B. amyloliquefaciens* inoculum under four water regimes (W100, W50, W25 and W0), with six independent tray-cell replicates per combination. We tested three hypotheses: (i) reduced water supply would dominate the seedling phenotype by increasing the leaf-surface temperature and restricting expansion and biomass accumulation; (ii) bacterial effects would be non-linear and water-regime-dependent, resulting in significant water × dose interactions rather than a universal optimum; and (iii) multivariate integration would distinguish coordinated whole-seedling phenotypes and provide a descriptive within-regime ranking without assuming a universal optimum.

## 2. Results

### 2.1. Water Regime Dominated Thermal and Architectural Responses

**Figure 1:**
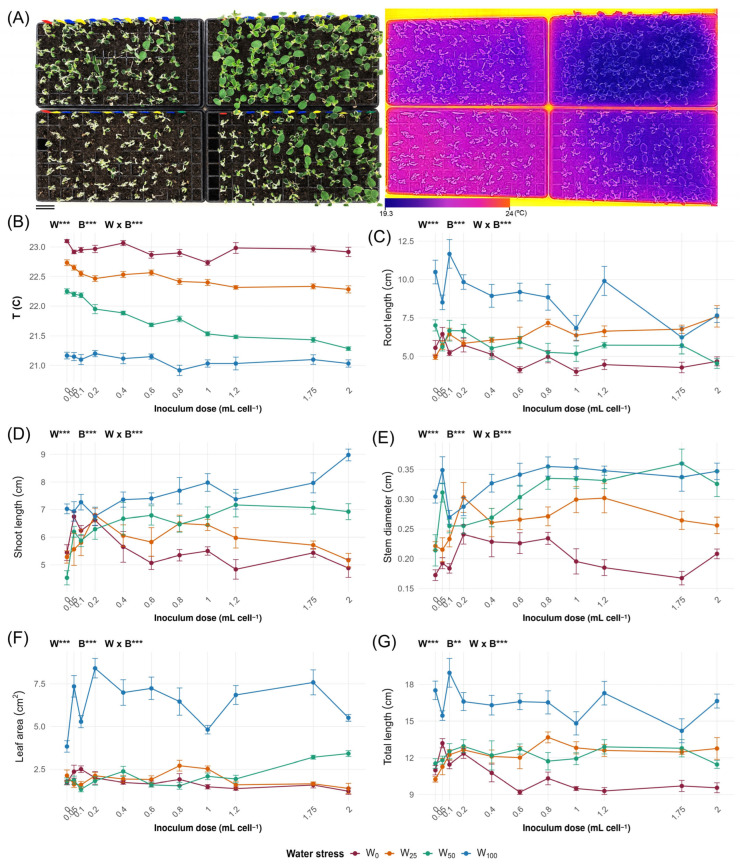
Thermal and architectural responses of soybean seedlings to water regime and *Bacillus subtilis* and *Bacillus amyloliquefaciens* inoculum dose. (**A**) Representative visible and radiometric images acquired at the end of the 15-day restriction period; these images illustrate canopy coverage and surface-temperature patterns and were not treated as additional replicates. Panels show (**B**) leaf-surface temperature (°C), (**C**) root length (cm), (**D**) shoot length (cm), (**E**) stem diameter (cm), (**F**) leaf area (cm^2^) and (**G**) total length (cm). The experiment comprised four water regimes × eleven inoculum doses × six independent tray-cell replicates (264 experimental units). Before water-regime imposition, each cell received one rhizosphere-directed application of an undiluted mixed *Bacillus subtilis* + *Bacillus amyloliquefaciens* suspension (5 × 10^8^ CFU mL^−1^) at 0, 0.05, 0.1, 0.2, 0.4, 0.6, 0.8, 1.0, 1.2, 1.75 or 2.0 mL cell^−1^; the 0 mL control received water without bacterial inoculum. During restriction, W100 was maintained at 100% operational field capacity, W50 and W25 received 50% and 25% of the volume required to restore W100, respectively, and W0 received no irrigation. Points are the means ± SEs (n = 6 independent cells). Lines connect ordered treatment means solely to aid visualisation and are not fitted by dose–response models. The labels above the panels report fixed effects from two-way ANOVA for water regime (W), inoculum dose (B) and W × B: ns (not significant), *p* ≥ 0.05; *, *p* < 0.05; **, *p* < 0.01; ***, *p* < 0.001. The radiometric scale spans 19.3–24.0 °C; the visible-image scale bar represents 4 cm.

Factorial ANOVA revealed that the water regime (W), inoculum dose (B) and the W × B interaction significantly affected the leaf-surface temperature, root length, shoot length, stem diameter, leaf area and total length (Figure 1B–G). For these traits, W and W × B were significant at *p* < 0.001; the dose effect was significant at *p* < 0.001, except for total length (*p* < 0.01). The visible and thermal-radiometric images were consistent with these model results: W0 seedlings had less canopy cover and warmer surfaces, whereas W100 seedlings had larger leaves and cooler surfaces (Figure 1A).

Averaged across inoculum doses, W0 increased the leaf-surface temperature by 8.8% relative to W100 (22.94 versus 21.09 °C) and reduced the root length by 44.3%, shoot length by 25.4%, stem diameter by 38.2%, leaf area by 72.3% and total length by 35.6% (Figure 1B–G). W50 and W25 generally occupied intermediate positions, confirming that the treatments generated an ordered water-supply gradient rather than a simple well-watered versus drought contrast. Leaf area was the most responsive architectural trait, followed by root length and stem diameter (Figures S1–S11).

The significant interactions reflected distinct dose profiles within each water regime (Figure 1B–G). Under W0, the greatest mean root and total length occurred at the lowest doses, especially 0.05 mL, whereas increasing the dose did not result in a progressive increase. Under W25, shoot length and stem diameter were most favourable near 0.2 mL, whereas total length peaked near 0.8 mL. Under W50, several intermediate-to-high doses improved specific traits, including shoot length at 1.2 mL and leaf area at 2.0 mL. Under W100, the untreated baseline was already high, and favourable responses were trait-specific rather than consistent across all endpoints (Figure S9).

Multiplicity-adjusted within-regime comparisons further illustrated this conditionality. Compared with that of the corresponding untreated control, the shoot length increased by 28.8% at 0.2 mL under W25 (*p* = 0.0116) and by 58.3% at 1.2 mL under W50 (*p* < 0.001). The leaf area increased by 89.8% at 2.0 mL under W50 (*p* = 0.0098) and by 119.4% at 0.2 mL under W100 (*p* < 0.001). In contrast, several visually positive W0 responses did not significantly change after multiplicity adjustment, indicating that strong water limitation increased replicate dispersion and reduced the resolution of some dose contrasts. The complete factorial-significance map, percentage changes and multiplicity-adjusted dose-versus-control estimates for these traits are provided in Supplementary Figures S1, S2, S5 and S9–S11.

### 2.2. Biomass Responses Depended on Organ, Dose and Water Regime

**Figure 2:**
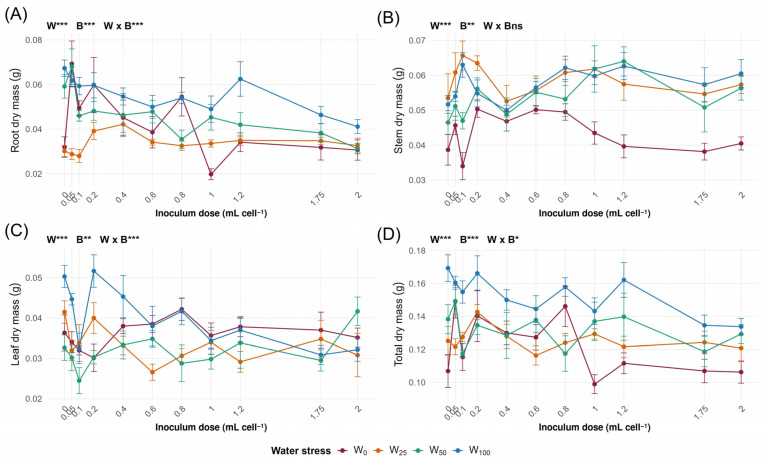
Dry mass responses of soybean seedlings to the water regime and *Bacillus subtilis* + *Bacillus amyloliquefaciens* inoculum dose: (**A**) root dry mass, (**B**) stem dry mass, (**C**) leaf dry mass and (**D**) total dry mass, all expressed as g plant^−1^. The four water regimes and eleven inoculum doses were imposed as described for Figure 1. The experiment included six independent tray-cell replicates per water × dose combination. Points are means ± SEs (n = 6), and connecting lines are visual guides rather than fitted dose–response curves. The labels above the panels report fixed effects from two-way ANOVA for water regime (W), inoculum dose (B) and W × B: ns (not significant), *p* ≥ 0.05; *, *p* < 0.05; **, *p* < 0.01; ***, *p* < 0.001. Abbreviation details are given in the caption of Figure 1 and Section 4.

The water regime significantly affected the root, stem, leaf and total dry mass, and the inoculum dose significantly affected all four biomass traits (Figure 2). The W × B interaction was significant for root dry mass, leaf dry mass and total dry mass; the stem dry mass interaction was not significant (*p* = 0.0552). Thus, water availability set the overall biomass ceiling, whereas the bacterial response differed among organs and water regimes.

Compared with W100, W0 reduced root dry mass by 23.2%, stem dry mass by 24.6% and total dry mass by 20.1% (Figure 2A,B,D). The leaf dry mass decreased by only 9.5%, which was markedly less than the 72.3% reduction in leaf area. This difference shows that severe water limitation changed leaf construction and expansion more strongly than it depleted the leaf biomass pool.

The clearest positive biomass response under W0 occurred at the lowest non-zero dose. Compared with the untreated W0 control, 0.05 mL increased root dry mass by 116.7% (*p* < 0.001) and total dry mass by 39.3% (*p* = 0.00368) (Figure 2A,D). Under W50, 1.2 mL increased the stem dry mass by 37.6% (*p* = 0.0121), whereas the greatest mean leaf dry mass occurred at 2.0 mL. For stem dry mass, the W × B interaction was not significant according to the classical Type III ANOVA (*p* = 0.0552), although the HC3 sensitivity analysis was significant (*p* = 0.0147); this organ-specific interaction was therefore interpreted cautiously. Under W100, no single dose consistently exceeded the untreated control across all four biomass compartments. Complete percentage-change and adjusted-contrast visualisations for biomass are provided in Supplementary Figures S3 and S6.

Collectively, the biomass data did not support a single rate recommendation. Low doses were most relevant to root and total dry mass under W0, whereas intermediate or high doses were favourable for particular organs when some water was available. These are treatment-specific seedling responses, but there is no evidence that increasing the inoculum volume necessarily increases the biomass, even though the partitioning of biomass is affected (Figure 2 and Figure S10).

### 2.3. Water Limitation Altered Biomass Partitioning and Leaf Construction

**Figure 3:**
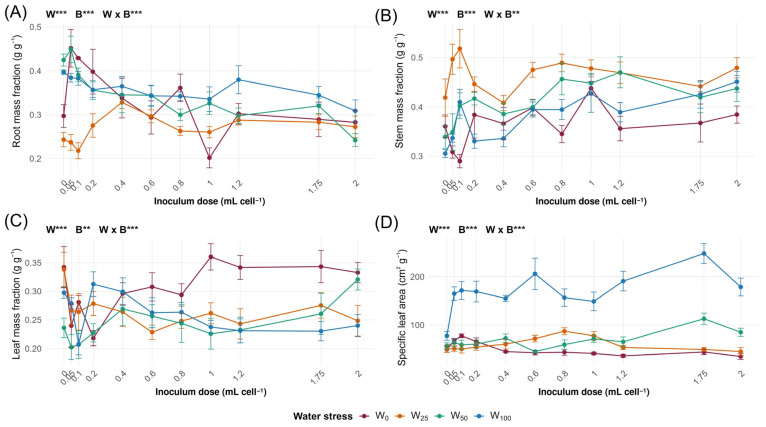
Biomass allocation and leaf construction in soybean seedlings: (**A**) root mass fraction (RMF = root dry mass/total dry mass), (**B**) stem mass fraction (SMF = stem dry mass/total dry mass), (**C**) leaf mass fraction (LMF = leaf dry mass/total dry mass) and (**D**) specific leaf area (SLA = leaf area/leaf dry mass). The mass fractions are expressed as g g^−1^, and the SLA is expressed as cm^2^ g^−1^. The seedlings received the four water regimes and eleven inoculum doses defined in Figure 1, with six independent tray cells per water × dose combination. Points are means ± SEs (n = 6); connecting lines aid visualisation only and are not fitted models. The labels above the panels report fixed effects from two-way ANOVA for water regime (W), inoculum dose (B) and W × B: ns (not significant), *p* ≥ 0.05; *, *p* < 0.05; **, *p* < 0.01; ***, *p* < 0.001. Details for the abbreviations are provided in the caption of Figure 1.

Water regime, inoculum dose and their interaction significantly affected the root mass fraction (RMF), stem mass fraction (SMF), leaf mass fraction (LMF) and SLA (Figure 3). The W × B term was significant at *p* < 0.001 for RMF, LMF and SLA and at *p* = 0.003 for SMF. These responses show that treatment effects involved proportional biomass allocation as well as absolute plant size.

Averaged across doses, W0 reduced SLA by 70.4% relative to W100 (50.19 versus 169.78 cm^2^ g^−1^) but increased LMF by 17.3% (Figure 3C,D). The combination of sharply lower SLA, much smaller leaf area and a relatively modest loss of leaf dry mass indicates that W0 seedlings produced less surface area per unit of leaf biomass. RMF and SMF changed less in the W0–W100 mean contrast, although both fractions remained responsive to the inoculum dose within individual water regimes.

Dose rankings differed across allocation traits. Under W0, 0.05 mL produced the highest RMF, whereas 1.0 mL produced the highest observed SMF and LMF means. SLA was highest at 1.75 mL under both W50 and W100, reaching 113.0 and 247.9 cm^2^ g^−1^, respectively (Figure 3). Thus, the inoculum could shift root investment, above-ground partitioning and leaf expansion efficiency in different directions depending on water availability. The corresponding percentage-change and adjusted-contrast visualisations are provided in Supplementary Figures S4, S7 and S11.

### 2.4. Thermal Status Covaried with Coordinated Growth Modules

**Figure 4:**
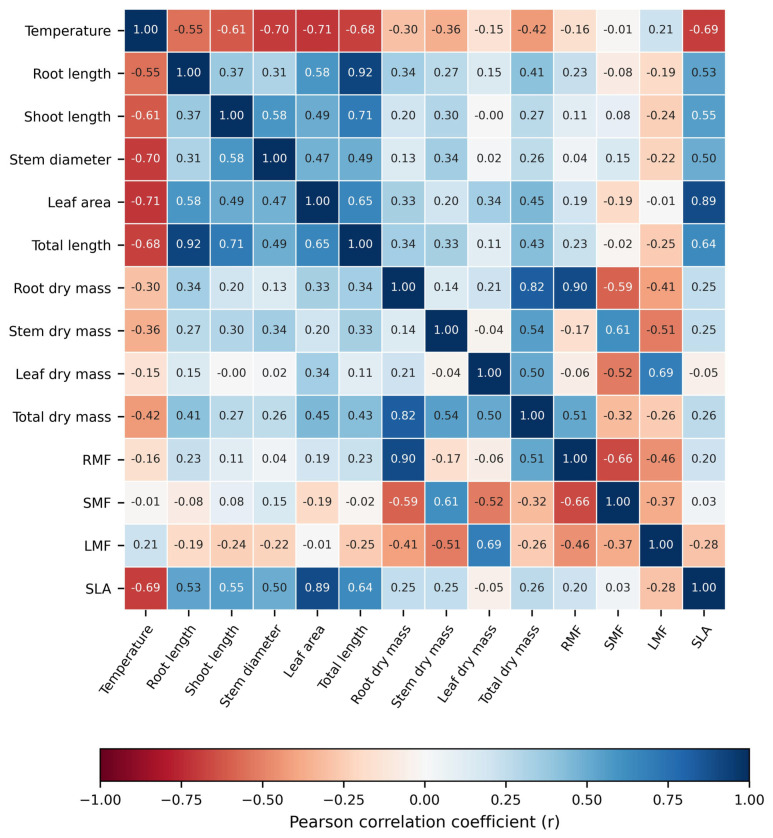
Pearson correlation matrix for the 14 measured or derived traits across all 264 independent tray-cell observations: leaf-surface temperature, root length, shoot length, stem diameter, leaf area, total length, root dry mass, stem dry mass, leaf dry mass, total dry mass, root mass fraction (RMF), stem mass fraction (SMF), leaf mass fraction (LMF) and specific leaf area (SLA). Each cell contains the Pearson correlation coefficient (r). Blue denotes positive associations, red denotes negative associations, and paler colours denote coefficients closer to zero. Correlations were calculated from replicate-level data pooled across the complete 4 × 11 factorial experiment.

Across the 264 biological replicates, leaf-surface temperature was negatively correlated with most expansion traits, including leaf area (r = −0.712), stem diameter (r = −0.703), SLA (r = −0.689), total length (r = −0.680), shoot length (r = −0.613) and root length (r = −0.549) (Figure 4). Warmer seedlings were therefore generally smaller and contained less leaf surface per unit of leaf mass.

Positive correlations defined complementary trait modules. Total length was strongly related to root length (r = 0.916) and shoot length (r = 0.710), leaf area was strongly related to SLA (r = 0.891), and root dry mass was related to both RMF (r = 0.897) and total dry mass (r = 0.823) (Figure 4). These relationships justified multivariate analysis but also indicated that several variables represented coordinated, partly redundant aspects of whole-seedling size and allocation.

### 2.5. Hierarchical Clustering Resolved Treatment-Level Phenotypes

**Figure 5:**
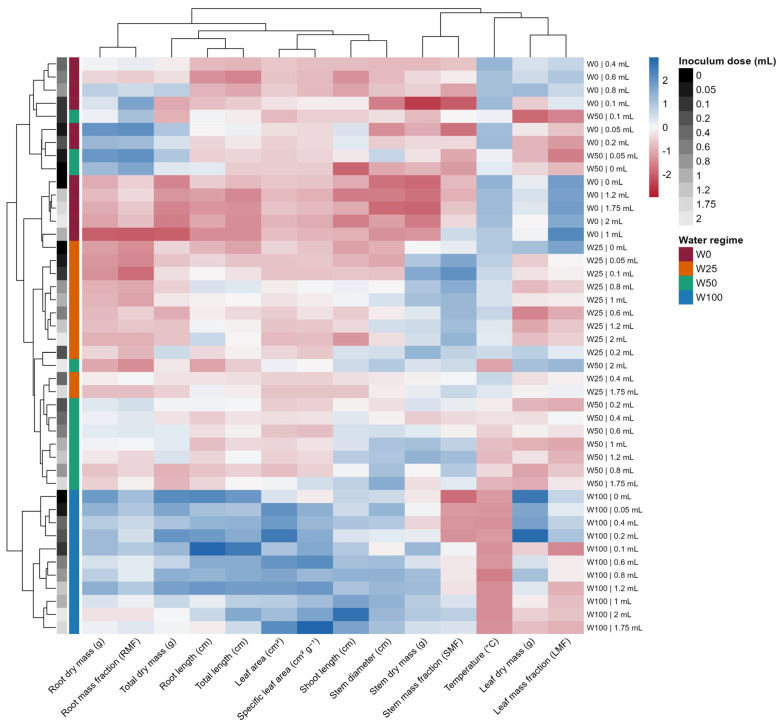
Hierarchical cluster heatmap of the 14-trait profiles for all 44 water-regime × bacterial-dose combinations. Each row represents one treatment-combination mean calculated from six independent tray cells, and each column represents a measured or derived trait. Before clustering, each trait was z-standardised across the 44 treatment means, producing a 44 × 14 matrix with one z-score for every treatment × trait combination; consequently, each treatment profile incorporated all 14 variables shown. Treatment rows and trait columns were clustered using Euclidean distance and complete-linkage agglomeration. The side bars identify inoculum dose (mL cell^−1^) and water regime, and the heatmap scale shows relative values for each trait: blue, above the across-treatment mean; red, below the mean; and white, near the mean. The water regimes, inoculum composition, dose sequence and 15-day restriction period were as defined in Figure 1.

Hierarchical clustering of the 44 water × dose treatments revealed separate combinations according to their standardised multivariate profiles (Figure 5). The W100 treatments resulted in relatively high leaf area, SLA, total length, root length, shoot length and stem diameter, whereas the W0 treatments resulted in relatively high temperature (blue colour) and low expansion (red colour). W25 and W50 occupied intermediate regions, although individual doses shifted their positions within the dendrogram (Figure 5). The z-standardised matrix contained one value for every treatment × trait combination, so each treatment profile incorporated all 14 variables shown in the heatmap (Figure 5).

The clustered profiles also confirmed that the dose responses were not monotonic (Figure 5). Several low-dose W0 combinations clustered closer to the intermediate-water treatments than did the higher-dose W0 combinations, which is consistent with the root- and total-dry-mass responses at 0.05 mL. Within W50, some intermediate-to-high doses were associated with greater stem diameter, total length or leaf expansion traits. The heatmap therefore reproduced the conditionality detected by factorial ANOVA without implying that proximity in the dendrogram constituted an independent significance test.

### 2.6. PCA Separated the Water Regimes and the Integrated Mean Z-Score Ranked Candidate Doses

**Figure 6:**
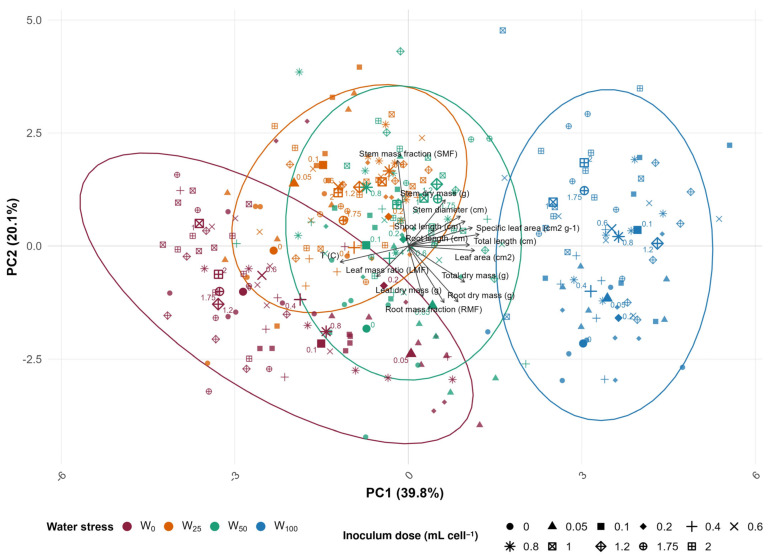
Principal component analysis of all 264 independent tray-cell observations. The 14 input variables were leaf-surface temperature, root length, shoot length, stem diameter, leaf area, total length, root dry mass, stem dry mass, leaf dry mass, total dry mass, root mass fraction (RMF), stem mass fraction (SMF), leaf mass fraction (LMF) and specific leaf area (SLA). Variables were centred and scaled to unit variance before analysis. Points represent biological replicates, colours identify the four water regimes, and symbols identify the eleven inoculum doses. PC1 and PC2 explain 39.8% and 20.1% of the total variance, respectively. Ellipses are 95% multivariate t-distribution data ellipses for each water regime. Larger symbols and numeric labels indicate the centroid of each dose within each water regime. Vectors indicate the direction and relative magnitude of trait loadings. Highly correlated and algebraically related variables were retained intentionally because the ordination was used descriptively to represent coordinated thermal, size and allocation modules, not to estimate independent causal effects. PCA was not used to calculate the within-regime dose ranking; that ranking was generated by the integrated index defined in Section 4.4 and the Supplementary Files.

The PCA of the centred and scaled replicate-level data summarised the dominant multivariate gradient (Figure 6). PC1 explained 39.8% of the total variance, and PC2 explained 20.1%, indicating that the cumulative variance was 59.9% (Figure 6). PC1 represented a vigour–water-limitation axis: total length, leaf area, SLA, root length, shoot length and total dry mass loaded in the positive direction, whereas leaf-surface temperature loaded in the opposite direction.

The mean PC1 scores were −2.18 for W0, −1.11 for W25, −0.09 for W50 and 3.39 for W100. W25 and W50 overlapped partly, indicating transitional phenotypes, whereas W0 and W100 occupied opposite regions of the ordination. Dispersion among symbols within each water-regime ellipse reflected dose-level modulation, but the water regime remained the dominant organiser of the multivariate structure (Figure 6).

The integrated mean z-score, calculated from ten thermal, architectural and biomass traits, was the sole multivariate summary used to rank doses within each water regime. It ranked 0.05 mL highest within W0, 0.2 mL within W25, 1.2 mL within W50 and 0.2 mL within W100; all cellwise scores and ranks are shown in Supplementary Figure S8. Correlation analysis, hierarchical clustering and PCA were used to describe the multivariate structure but not to generate these rankings. Because the score is an equal-weight average of standardised responses and does not generate *p*-values, the candidate rankings must be interpreted together with the trait-level ANOVA and multiplicity-adjusted contrasts shown in Supplementary Figures S1–S7.

## 3. Discussion

### 3.1. Water Limitation Constrained Evaporative Cooling and Organ Expansion

The first hypothesis was supported: the water regime imposed a dominant constraint on seedling performance. W0 plants were relatively warm and exhibited the greatest restrictions in terms of leaf area, root length, stem diameter, total length and SLA, whereas W25 and W50 generally occupied intermediate positions. This coordinated response is consistent with general drought physiology involving turgor loss, hydraulic limitation, stomatal regulation, stress signalling and growth–defence trade-offs [1,2,3,4,5,6]. Root hydraulics, architectural plasticity [30,31,32,33] and phenotyping frameworks [34,35], explain why the response was expressed simultaneously in terms of elongation, leaf deployment and biomass allocation [30,31,32,33,34,35]. Importantly, the decrease in leaf area was much greater than the decrease in total or leaf dry mass. Therefore, the way in which the biomass was deployed changed before all the biomass pools were proportionally depleted [36,37]. Recent studies in soybean plants under progressive or field drought reinforce the need to validate these early responses against genotype- and environment-dependent agronomic performance [7,8].

Leaf-surface temperature in seedlings provides an integrative, non-destructive indicator of this water-limitation syndrome rather than a direct measurement of any single physiological process [38,39]. Reduced transpiration can diminish evaporative cooling, but radiometric temperature is also influenced by canopy size, leaf angle, boundary-layer conditions, emissivity and the proportion of the plant surface within the image [38,39]. The negative associations of temperature with leaf area, stem diameter, total length and SLA are therefore biologically coherent, yet they should not be interpreted as proof that stomatal closure alone caused the growth response. Thermal imaging nevertheless strengthened phenotypic discrimination by linking warmer surfaces to a compact, low-expansion phenotype, whereas the broader drought-smart soybean literature supports combining such rapid phenotyping with physiological and agronomic validation [34,35,38,39,40].

### 3.2. Drought Promoted Conservative Leaf Construction and Reconfigured Allocation

The concurrent 17.3% increase in LMF and 70.4% decrease in SLA under W0 provide the clearest evidence that water limitation altered leaf construction rather than simply reducing all organs in parallel. LMF describes the fraction of total plant dry mass retained in leaves, whereas SLA describes the leaf surface deployed per unit of leaf dry mass [36,37]. Together with the 72.3% decrease in leaf area but only a 9.5% decrease in leaf dry mass, these values show that W0 seedlings retained a substantial proportion of their investment in leaves while producing far less surface from that investment. Because SLA is the inverse of leaf mass per area, the pattern is consistent with more mass-intensive, conservative leaves that may limit the exposed transpiring area and increase structural investment per unit surface [36,37].

Allocation ratios must be interpreted together with their component masses because a fraction can change through variation in either its numerator or total plant mass [37]. In the present dataset, the *Bacillus* treatment affected the RMF, SMF, LMF and SLA as well as the organ dry mass, indicating that the response was not reducible to a uniform increase in plant size. The 0.05 mL W0 treatment combined greater root dry mass with the highest RMF, whereas the other treatments shifted aboveground allocation or SLA when more water was available. This pattern is compatible with the ability of the inoculum to act as a water-dependent modifier of carbon deployment and organ balance rather than as a universal growth promoter [17,18,19,21,26,27,28]. Context-dependent crop responses and established PGPR mechanisms provide plausible routes involving root growth, nutrient acquisition [13,14,20,21,22,23,24,26], stress-ethylene regulation [20,21,23,25], microbial persistence [10,15,21,24], and consortium behaviour [18,19,28,29]. The defensible conclusion is therefore narrower: the inoculum dose changed the realised allocation phenotype, and the direction of that change depended on the water regime.

### 3.3. Bacterial Effects Were Nonlinear and Conditional on Water Availability

The widespread W × B interactions and the shift in the highest-ranked dose among W0, W25, W50 and W100 support the second hypothesis and exclude a universal dose optimum within this experiment. These results are consistent with recent evidence that microbial benefits depend on both biological treatment and the environmental context [9,10,11,12,20,21,27,28]. In a two-year dryland-maize study, Abrar et al. [17] reported that the effects of AM fungi and PGPR on hormones, photosynthesis and nutrient partitioning varied among developmental stages. Rehman et al. [18] evaluated *Rhizophagus irregularis* with *Bacillus amyloliquefaciens* in wheat at 80%, 50% and 35% field water capacity and reported water level-dependent changes in rhizosphere activity, carbon efficiency and crop performance. Khan et al. [19] similarly tested *Acaulospora laevis* with *Bacillus subtilis* in maize at 80%, 55% and 35% water levels and linked the combined treatment to root–leaf function across moisture conditions. These studies used different crops, defined strains and AM–fungal–bacterial coinoculations; thus, they did not validate the dose values observed here. They do, however, provide independent support for the central principle that microbial performance cannot be separated from crop stage and water availability [9,10,11,12,17,18,19,21,27,28].

Several processes could generate a nonlinear dose response. *Bacillus* endospores can support formulation persistence [21,22], while rhizosphere-competent strains may influence nutrient mobilisation [20,21,22,23,24], auxin- and ethylene-related signalling [20,21,22,23,25], extracellular polysaccharide activity, biofilm formation, osmotic adjustment and antioxidant protection [22,23,24,26]. The expression of these functions depends on viable cell density, root exudation, substrate diffusion and water films that permit microbial activity and metabolite transport [9,10,11,12,15,21,24,29]. Under W0, a small inoculum may have been sufficient to precondition the initially moist rhizosphere, after which the progressive hydraulic constraint limited the additional plant response [21,23,24,26]. Under W50, greater residual plant and microbial activity may have allowed higher inoculum volumes to influence selected traits [17,18,19]. These explanations remain open and should be tested directly.

At the plant level, any microbial effect operates against the background of endogenous abiotic stress signalling [1,2,3,4]. Protein-kinase networks [41], reactive oxygen sensing [42], calcium transport [43], cyclic nucleotide-gated channels [44,45], thermosensing [46,47], and osmolyte metabolism [48] are established components of plant responses to environmental stress.

### 3.4. Multivariate Integration Strengthened, but Did Not Replace, Trait-Level Inference

The third hypothesis was supported by convergence among the correlation matrix, hierarchical clustering, PCA and the integrated score. All four approaches resolved the same dominant gradient: cool, expansive and comparatively vigorous W100 seedlings contrasted with warm, compact W0 seedlings, whereas W25 and W50 occupied transitional positions. This convergence is biologically useful because water limitation is expressed through coordinated changes in root and shoot extension and root hydraulic architecture [30,31,32,33], phenotypic integration [34,35], leaf deployment and biomass allocation [36,37], and thermal status [38,39], rather than through one isolated variable. This finding also explains why no single trait can define the best bacterial treatment. A dose may improve one organ or allocation fraction without moving the entire phenotype in the same direction, whereas another may produce a moderate but coordinated change across several traits.

The multivariate outputs complemented, but did not replace, the factorial inference. ANOVA and multiplicity-adjusted contrasts were used to test treatment effects. The correlation matrix, hierarchical clustering and PCA described covariation and treatment structure, whereas the integrated mean z-score alone generated the within-regime ranking reported in Section 2.6.

### 3.5. Practical Significance, Study Scope and Priorities for Agronomic Validation

This study provides a factorially resolved framework for identifying *Bacillus* doses with the greatest potential to support early soybean growth under contrasting levels of water availability. Importantly, the results indicate that biological performance cannot be represented by a single dose applied uniformly across water conditions; instead, the most favourable responses emerged from specific dose–water combinations [17,18,19,21,26,27,28]. The candidate doses identified here therefore represent technically supported starting points for the next stages of agronomic validation [21,27]. A logical progression would include experiments in larger rooting volumes with continuous monitoring of substrate water content, followed by evaluation across contrasting soil textures, organic-matter levels and resident microbial communities [9,10,11,12,15,24,29]. Application strategy should also be optimised by comparing seed treatment, in-furrow delivery, application before or during water limitation and repeated delivery, while assessing formulation viability and rhizosphere persistence [21,22,24,27]. Because commercial soybean production is closely associated with *Bradyrhizobium*-based inoculation, compatibility with standard nodulation practices should be incorporated into this validation process. Subsequent multienvironment trials can then determine whether the early growth responses observed here translate into improved establishment, nodulation, root development, plant water relations, reproductive performance, yield stability and economic return [7,8,17,18,19,26,40].

The controlled tray system was intentionally used as a high-resolution screening platform, enabling the effects of water regime, inoculum dose and their interaction to be examined under standardised conditions and with independent biological replication. The resulting conclusions should therefore be interpreted within the defined experimental domain: early soybean establishment, a 15-day evaluation period, one cultivar, one mixed-species biological formulation and a single preconditioning protocol. W50 and W25 represented operational fractions of the irrigation volume supplied to W100, whereas W0 constituted an intentionally severe water-withholding treatment initiated in previously moist substrate; these treatments provided a reproducible gradient of water availability but were not intended to reproduce every temporal and spatial feature of field drought [1,6,7,8,40]. The experiment was designed primarily to resolve phenotypic and growth responses, rather than to establish the underlying physiological, molecular or microbiological mechanisms. Measurements of colonisation dynamics [9,10,11,12,15,21,24,29], gas exchange, plant water status, photosynthetic performance, hormone and redox regulation, osmotic adjustment [1,2,3,4,7,20,21,22,23,26,41,42,48], nodulation and yield [8,17,18,19,26,40] will be valuable for determining how the observed responses arise and whether they persist throughout the crop cycle. Nevertheless, the defined experimental scope does not diminish the central finding: water availability was the principal determinant of the soybean seedling phenotype, while the direction and magnitude of the bacterial effect were jointly governed by dose and water regime. This interaction provides a clear experimental basis for selecting treatments for mechanistic investigation and agronomic validation [21,27,40].

## 4. Materials and Methods

### 4.1. Plant Material and Controlled-Environment Conditions

Soybean (*Glycine max* (L.) Merr.) seeds of cultivar BRS 1056 IPRO were obtained from Embrapa Soybean (Londrina, Paraná, Brazil). The study involved a controlled-environment tray-cell experiment, not a field trial. The seedlings were grown at 25 ± 2 °C and 60 ± 10% relative humidity under a 16 h photoperiod and a photosynthetic photon flux density of approximately 500 µmol photons m^−2^ s^−1^. Each substrate-filled tray cell functioned as a small pot and constituted the experimental unit.

Before water regime implementation, all cells were managed under the same water supply to standardise establishment. During this common-establishment period, all the seedlings received the same volume (5 mL) of complete Hoagland nutrient solution weekly, thereby limiting nutritional imbalance among the treatments. *Bradyrhizobium* was not applied; the deliberately manipulated rhizosphere factor was the mixed *Bacillus* treatment. The selected formulation contained the two microorganisms as a co-cultivated mixture.

### 4.2. Experimental Design, Bacterial Preconditioning and Water Regimes

The experiment followed a completely randomised 4 × 11 factorial design with four water regimes, eleven inoculum doses and six biological replicates per combination (264 independent cells). The inoculum was an undiluted suspension containing *Bacillus subtilis* + *Bacillus amyloliquefaciens* at 5 × 10^8^ colony-forming units (CFUs) mL^−1^. The source records identified the organisms to the species level but did not contain strain codes; consequently, the conclusions about this mixed-species formulation, not strain-specific performance, are supported. A single rhizosphere-directed application supplied 0, 0.05, 0.1, 0.2, 0.4, 0.6, 0.8, 1.0, 1.2, 1.75 or 2.0 mL cell^−1^. These treatments are referred to as inoculum doses because the suspension concentration was constant and the applied volume varied. The 0 mL controls received water without bacterial inoculum.

The inoculum was applied before water restriction to ensure that initial plant establishment was common to all treatments and that the suspension could contact the rhizosphere before drought developed. This sequence represents prophylactic rhizosphere preconditioning and does not include seed treatment, application during established drought or repeated application. Substrate field capacity was calibrated gravimetrically after saturation and free drainage. During the subsequent 15-day restriction period, W100 cells were maintained at 100% operational field capacity; W50 and W25 received 50% and 25%, respectively, of the water volume required to restore W100; and W0 received no additional water. W50 and W25 therefore denote replacement fractions, not fixed measurements of 50% and 25% volumetric water content. W0 was included as a severe end-member: plants entered the restriction period with stored substrate water and progressively depleted it rather than being established in an initially dry substrate. Trays were periodically repositioned to reduce positional effects.

### 4.3. Growth, Biomass Allocation and Thermal Imaging

At the end of the restriction period, the root length (RL), shoot length (SL), stem diameter and total leaf area (LA) of the same plants were measured. The detached leaflets were scanned and analysed with ImageJ version 1.54 s (National Institutes of Health, Bethesda, MD, USA). Images were spatially calibrated using a reference scale included in each scan. The plants were separated into roots, stems and leaves, and the tissues were dried in a forced-air oven at 70 °C to a constant mass. Root dry mass (RDM), stem dry mass (SDM), leaf dry mass (LDM) and total dry mass (TDM) were expressed as g plant^−1^. Total length (TL), dry-mass fractions and SLA were calculated according to established leaf-construction and biomass-allocation definitions [36,37], as follows:

TDM=RDM+SDM+LDM,    TL=RL+SL

$$RMF=\frac{RDM}{TDM},\;\;SMF=\frac{SDM}{TDM},\;\;LMF=\frac{LDM}{TDM},\;\;SLA=\frac{LA}{LDM}$$

SLA was expressed as cm^2^ g^−1^, and RMF, SMF and LMF were expressed as g g^−1^. Radiometric images were acquired from intact plants with a FLIR EDGE Pro thermal camera (FLIR Systems, Danderyd, Sweden; 7.5–13.5 µm spectral band) positioned perpendicular to the canopy at a fixed distance. Leaf emissivity was set to 0.98, following the protocol previously applied to soybean under progressive water deficit [7]. Thermal image acquisition and interpretation followed established recommendations for plant-stress phenotyping [38,39].

### 4.4. Statistical Analysis and Multivariate Integration

The data are reported as the means ± standard errors (SEs), with six independent biological replicates per water × dose cell. Water regime (W100, W50, W25 and W0), inoculum dose (0, 0.05, 0.1, 0.2, 0.4, 0.6, 0.8, 1.0, 1.2, 1.75 and 2.0 mL) and W × B were treated as fixed effects in Type III two-way ANOVA. Doses were categorical in the primary factorial analysis and ordered only for graphical display. Residual normality and heteroscedasticity were examined using the Shapiro–Wilk and Breusch–Pagan tests, respectively [49,50]. Type III tests used sum-to-zero contrasts for the categorical factors, and heteroscedasticity-robust tests (HC3) were performed as a sensitivity analysis:

$${\hat{Var}}_{HC3}\left(\overset{ˆ}{\beta }\right)={\left(X^{\prime }X\right)}^{-1}X^{\prime }diag\left[\frac{e_{i}^{2}}{{\left(1-h_{ii}\right)}^{2}}\right]X{\left(X^{\prime }X\right)}^{-1}$$

where ${\hat{Var}}_{HC3}\left(\hat{\beta }\right)$ is the HC3 heteroscedasticity-consistent covariance matrix of the estimated regression coefficients; $\hat{\beta }$ is the vector of estimated model coefficients; X is the model matrix; $X^{\prime }$ denotes the transpose of X; ${\left(X^{\prime }X\right)}^{-1}$ is the inverse of the cross-product matrix; $e_{i}$ is the ordinary least-squares residual for observation i; $h_{ii}$ is the leverage of observation i, corresponding to the ith diagonal element of the hat matrix $H=X{\left(X^{\prime }X\right)}^{-1}X^{\prime }$; and $diag\left[\cdot \right]$ denotes a diagonal matrix whose ith element is $e_{i}^{2}/{\left(1-h_{ii}\right)}^{2}$. The factor ${\left(1-h_{ii}\right)}^{-2}$ increases the contribution of residuals from high-leverage observations, providing heteroscedasticity-robust standard errors, confidence intervals and Wald tests.

Tukey adjustment was used for all pairwise estimated-mean comparisons, whereas the planned contrasts of each nonzero dose against the corresponding 0 mL control within a water regime used the Holm multiplicity adjustment. The figure codes ns, *, ** and *** denote non-significance, *p* ≥ 0.05, *p* < 0.05, *p* < 0.01 and *p* < 0.001, respectively [49,50]. Model-wide significance, percentage changes, adjusted within-regime contrasts and the integrated ranking are visualised in Supplementary Figures S1–S11. Residual diagnostics, effect estimates, confidence intervals and multiplicity-adjusted *p*-values were interpreted jointly rather than using *p*-values as the sole basis for inference [50].

The integrated score was not an additional hypothesis test. It condenses ten measured responses into one descriptive mean z-score solely to rank treatment combinations within each water regime; this is the calculation used for the rankings reported in the Abstract, Section 2.6 and Section 5. For treatment combination j and trait k, the treatment-cell mean was standardised across the 44 water × dose combinations:

$$z_{jk}\;=\;\frac{\bar{x_{jk}}\;-\;\mu_{k}}{s_{k}}$$

In this expression, the numerator is the difference between the treatment-cell mean and the across-cell mean for trait k, and the denominator is the standard deviation of the 44 treatment-cell means for that trait. Ten traits were included: leaf-surface temperature, root length, shoot length, stem diameter, leaf area, total length, root dry mass, stem dry mass, leaf dry mass and total dry mass. The script-defined integrated mean z-score was then calculated as follows:

$$I_{j}\;=\;\frac{1}{10}\;\Sigma_{k\;=\;1}^{10}\;z_{jk}$$

All ten standardised traits contributed equally, and the leaf-surface temperature was retained in its observed direction, as implemented in the analysis script. Thus, a higher score denotes a higher arithmetic mean of the ten standardised responses; it is not, by itself, a universal physiological-benefit score. Scores were ranked in descending order separately within W0, W25, W50 and W100, and interpretation was checked against the direction and significance of the individual traits. The manuscript ranking used this mean z-score (the analysis output is labelled mean_z_score) [49,50]. A separately exported min–max summary was not used to determine candidate doses or statistical significance. The complete scores and within-regime ranks are provided in Supplementary Figure S8.

PCA used all 264 replicates and 14 centred and unit-variance-scaled variables: leaf-surface temperature, root length, shoot length, stem diameter, leaf area, total length, root, stem, leaf and total dry mass, RMF, SMF, LMF and SLA. Highly correlated and algebraically related variables were retained intentionally because PCA was used descriptively to represent the coordinated whole-seedling phenotype; loadings were interpreted as trait modules rather than independent causal effects. The cluster heatmap used the 44 treatment-cell means for the same 14 variables, including leaf-surface temperature. For each trait, the 44 treatment means were z-standardised, producing one z-score for every treatment × trait cell in the 44 × 14 matrix; Euclidean distance and complete-linkage clustering were then applied to both treatment rows and trait columns [49,50]. The Pearson correlations were calculated using replicate-level observations.

**Figure 7:**
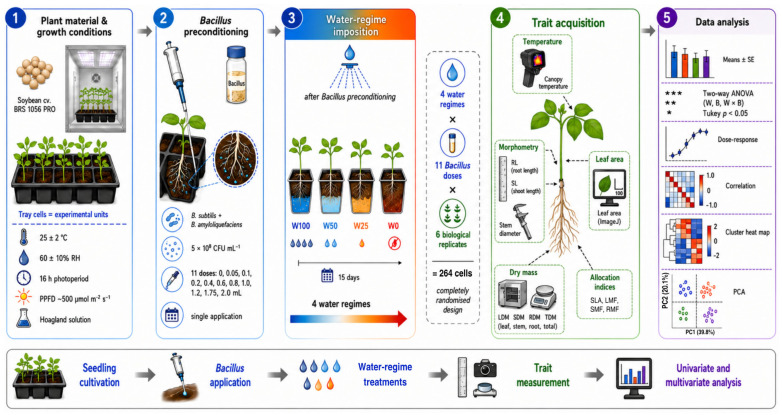
Experimental workflow for the controlled-environment soybean tray-cell assay. Seedlings of cv. BRS 1056 IPRO were assigned to a completely randomised 4 × 11 factorial design comprising W100, W50, W25 and W0 water regimes and one rhizosphere-directed application of an undiluted *Bacillus subtilis* + *Bacillus amyloliquefaciens* suspension (5 × 10^8^ CFU mL^−1^) at 0, 0.05, 0.1, 0.2, 0.4, 0.6, 0.8, 1.0, 1.2, 1.75 or 2.0 mL cell^−1^. Each combination included six independent tray cells, totalling 264 experimental units. Bacterial preconditioning preceded the 15-day restriction period. W100 was maintained at 100% operational field capacity; W50 and W25 received 50% and 25% of the W100 replacement volume, respectively; and W0 received no irrigation. Thermal, architectural, leaf-area, organ dry-mass and allocation traits were tested by two-way ANOVA and adjusted comparisons. Pearson correlation, hierarchical clustering and PCA described multivariate structure; the integrated mean z-score generated the descriptive within-regime dose ranking; and exploratory regression screening was not used for inference or dose ranking. Asterisks indicate the level of statistical significance: ***, *p* < 0.001; **, *p* < 0.01; *, *p* < 0.05, based on the Two-way ANOVA for (W, B, W × B) and Tukey post-hoc significance. Supplementary Figures S1–S11 provide the full significance map, percentage changes, adjusted contrasts and integrated-score visualisation. Created with BioRender.com.

Exploratory within-regime regression models were fitted during data screening to examine broad linear and non-linear tendencies across the ordered doses. They are not reported because the primary inferential scope treated dose as a categorical factor in the 4 × 11 design and the regression output did not add a distinct biological or statistical conclusion. These models were not used to assign significance, replace the factorial ANOVA or determine the candidate-dose rankings reported in Section 2.6. Analyses and graphics were produced in R version 4.5.2 (R Foundation for Statistical Computing, Vienna, Austria) using RStudio version 2026.04 (Posit PBC, Boston, MA, USA), Statistica version 12.0 (TIBCO Software Inc., Palo Alto, CA, USA) and SigmaPlot version 15.0 (Systat Software Inc., San Jose, CA, USA) (Figure 7).

## 5. Conclusions

In this controlled-environment study, the fully crossed 4 × 11 factorial design, comprising 264 independent tray-cell experimental units, demonstrated that water availability not only dominated soybean seedling performance but also determined the direction and magnitude of the response to *B. subtilis* + *B. amyloliquefaciens* preconditioning. Averaged across inoculum doses, W0 increased leaf-surface temperature by 8.8% and reduced root length by 44.3%, shoot length by 25.4%, stem diameter by 38.2%, leaf area by 72.3%, total dry mass by 20.1% and SLA by 70.4% relative to W100. The concurrent 17.3% increase in LMF revealed a marked shift in biomass allocation: severely water-restricted seedlings retained a greater proportion of biomass in leaves while deploying substantially less leaf surface per unit leaf mass.

Crucially, the bacterial response was non-monotonic, trait-specific and strongly conditioned by water regime, demonstrating that a greater inoculum dose did not necessarily produce a greater biological response. Under W0, 0.05 mL cell^−1^ was the clearest biomass-enhancing treatment, increasing root dry mass by 116.7% and total dry mass by 39.3% relative to the untreated W0 control. The integrated response ranking further prioritised 0.05 mL cell^−1^ under W0, 0.2 mL cell^−1^ under W25, 1.2 mL cell^−1^ under W50 and 0.2 mL cell^−1^ under W100. These combinations therefore provide an evidence-based shortlist for targeted pre-drought screening rather than supporting a single inoculum dose across contrasting water conditions.

Direct agronomic extrapolation remains constrained by the tray-cell environment, single soybean cultivar and mixed formulation, unavailable strain codes, single preconditioning application and absence of colonisation, nodulation and yield measurements. Nevertheless, the study provides a high-resolution experimental map of the water regime × inoculum dose interaction across thermal, architectural, biomass and allocation traits. It establishes water availability as an essential criterion for selecting microbial treatments, shows that a low inoculum dose can enhance biomass accumulation under severe water restriction, and provides a strong experimental basis for mechanistic, larger-pot and field validation.

## Data Availability

The processed data and statistical outputs supporting the findings of this study are provided in the article and its Supplementary Materials.

## Supplementary Materials

The following supporting information can be downloaded at: https://www.mdpi.com/article/10.3390/plants15152267/s1, Figure S1, model-wide Type III ANOVA significance across all 14 response variables; Figures S2–S4, percentage changes relative to the corresponding 0 mL control within each water regime; Figures S5–S7, Holm-adjusted inoculum-dose contrasts against 0 mL with 95% confidence intervals; Figure S8, integrated mean z-scores and within-regime dose ranks; and Figures S9–S11, Tukey-adjusted all-pairwise inoculum-dose groupings for thermal and architectural, dry-mass, biomass-allocation and specific-leaf-area traits. These materials provide a complete graphical statistical complement to Figure 1, Figure 2, Figure 3, Figure 4, Figure 5 and Figure 6.
