# Supplementary material for: Water Regime Determines Dose-Dependent Growth and Biomass Allocation Responses of Soybean Seedlings to Bacillus subtilis and Bacillus amyloliquefaciens
Source: Plants (Basel). 2026 Jul 24;15(15):2267. doi: 10.3390/plants15152267 (PMC13467629; doi:10.3390/plants15152267)
Supplement: Supplementary file 1 [file plants-15-02267-s001.zip › plants-4449105-supplementary.pdf]

Supplementary Materials

Water Regime Determines Dose-Dependent Growth and Biomass Allocation Responses of Soybean Seedlings to *Bacillus subtilis* and *Bacillus amyloliquefaciens*

João Paulo Alves da Silva [1], Luis Guilherme Teixeira Crusiol [2], José Salvador Simoneti Foloni [2], José Renato Bouças Farias [2], Marcelo Luiz Chicati [1], Roney Berti de Oliveira [1], Renato Herrig Furlanetto [3], José A. M. Demattê [4], Marcos Rafael Nanni [1] and Renan Falcioni [1,2,*]

[1] Graduate Program in Agronomy, State University of Maringá, Av. Colombo, 5790, Maringá 87020-900, PR, Brazil; ra125202@uem.br (J.P.A.d.S.); mlchicati@uem.br (M.L.C.); rboliveira@uem.br (R.B.d.O.); mrnanni@uem.br (M.R.N.)

[2] National Soybean Research Center, Brazilian Agricultural Research Corporation, Rodovia Carlos João Strass, s/n°, Distrito de Warta, Londrina 86001-970, PR, Brazil; luis.crusiol@colaborador.embrapa.br (L.G.T.C.); salvador.foloni@embrapa.br (J.S.S.F.); joserenato.farias@embrapa.br (J.R.B.F.)

[3] Gulf Coast Research and Education Center, University of Florida, Wimauma, FL 33598, USA; re.herrigfurlane@ufl.edu

[4] Department of Soil Science, Luiz de Queiroz College of Agriculture, University of São Paulo, Av. Pádua Dias, 11, Piracicaba 13418-260, SP, Brazil; jamdemat@usp.br

* Correspondence: renanfalcioni@gmail.com or rfalcioni2@uem.br; Tel.: +55-44-30118940

Supplementary statistical methods

The experiment included four water regimes (W100, W50, W25 and W0), eleven *Bacillus subtilis* + *Bacillus amyloliquefaciens* inoculum doses (0, 0.05, 0.1, 0.2, 0.4, 0.6, 0.8, 1.0, 1.2, 1.75 and 2.0 mL cell^{-1}) and six independent tray-cell replicates per combination (n = 264). Type III two-way ANOVA was used to analyse the water regime (W), bacterial dose (B) and W × B as fixed effects. Tukey adjustment was used for all-pairwise estimated-mean comparisons, whereas the planned contrasts of each non-zero dose against 0 mL within the same water regime used the Holm adjustment. Forest plots show estimated differences and 95% confidence intervals; red symbols indicate adjusted p < 0.05. The percentage changes were calculated from the corresponding treatment-cell means as follows:

$$\Delta\,(\%) = ((\bar{x}_d - \bar{x}_0)/\bar{x}_0) \times 100$$

The integrated ranking used treatment-cell means for ten traits: leaf-surface temperature, root length, shoot length, stem diameter, leaf area, total length, root dry mass, stem dry mass, leaf dry mass and total dry mass. Each trait was standardised across the 44 water × dose means, and the resulting z-scores were averaged with equal weight:

$$z_{jk} = \frac{\overline{x_{jk}} - \mu_k}{s_k}$$

$$I_j = \frac{1}{10}\,\Sigma_{k=1}^{10}\, z_{jk}$$

The leaf surface temperature was retained in its observed direction, as implemented in the analysis in RStudio version 2026.04. The score is therefore a descriptive composite response summary rather than an independent test or universal physiological-benefit metric. Doses were ranked in descending order separately within each water regime, and the rankings were interpreted together with the ANOVA and adjusted contrasts.

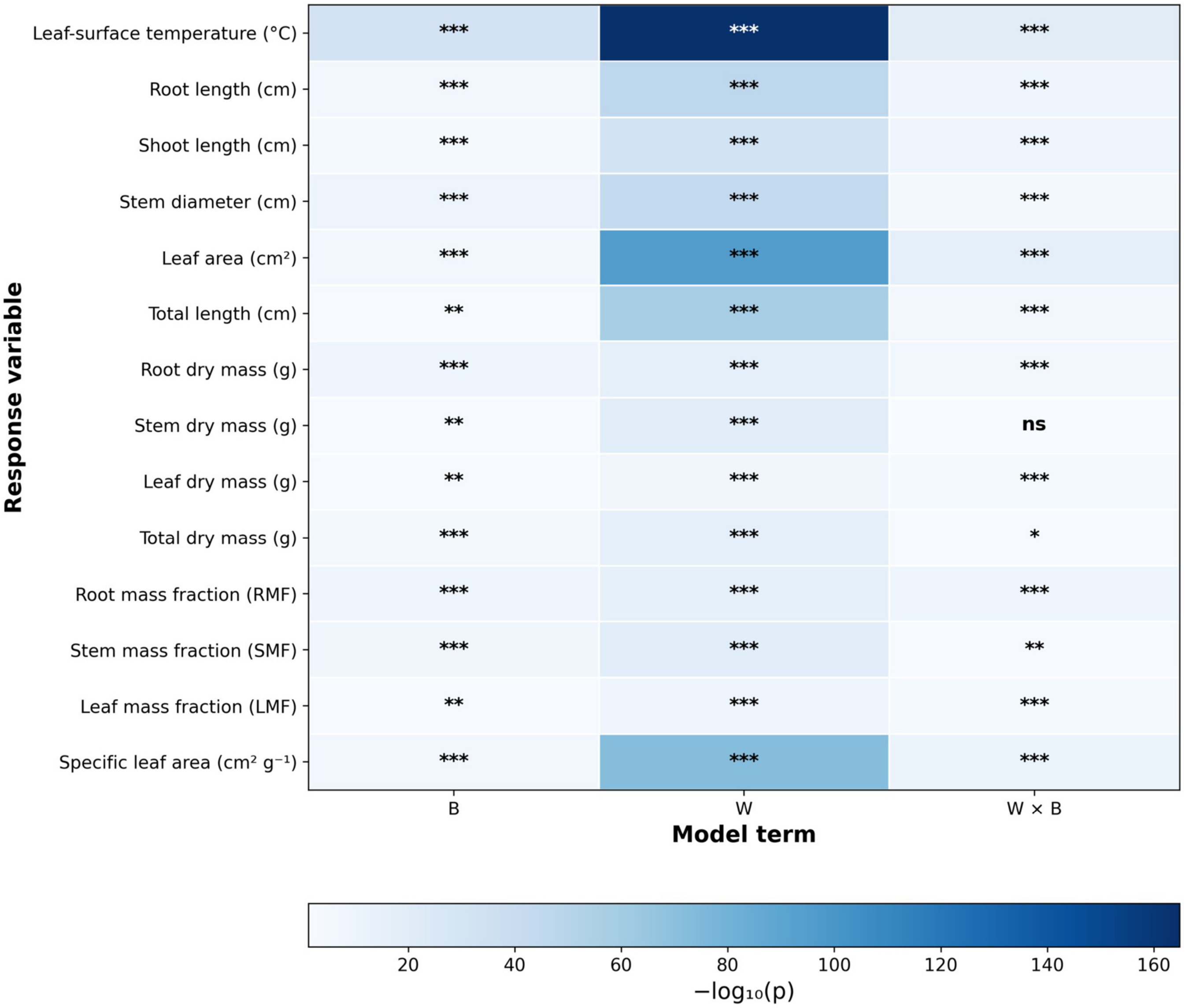

Figure S1. Global significance map for the factorial effects of bacterial dose (B), water regime (W) and their interaction (W × B) across the 14 measured or derived soybean seedling responses. Cell colour represents −log10(p) from the Type III two-way ANOVA; darker cells indicate smaller p-values. Symbols denote ns (not significant), $p \geq 0.05$; *, $p < 0.05$; **, $p < 0.01$; and ***, $p < 0.001$. The design comprised four water regimes × eleven doses × six independent tray cells (264 experimental units). Dry-mass variables are expressed as g plant^{-1}, mass fractions as g g^{-1} and SLA as cm^2 g^{-1}.

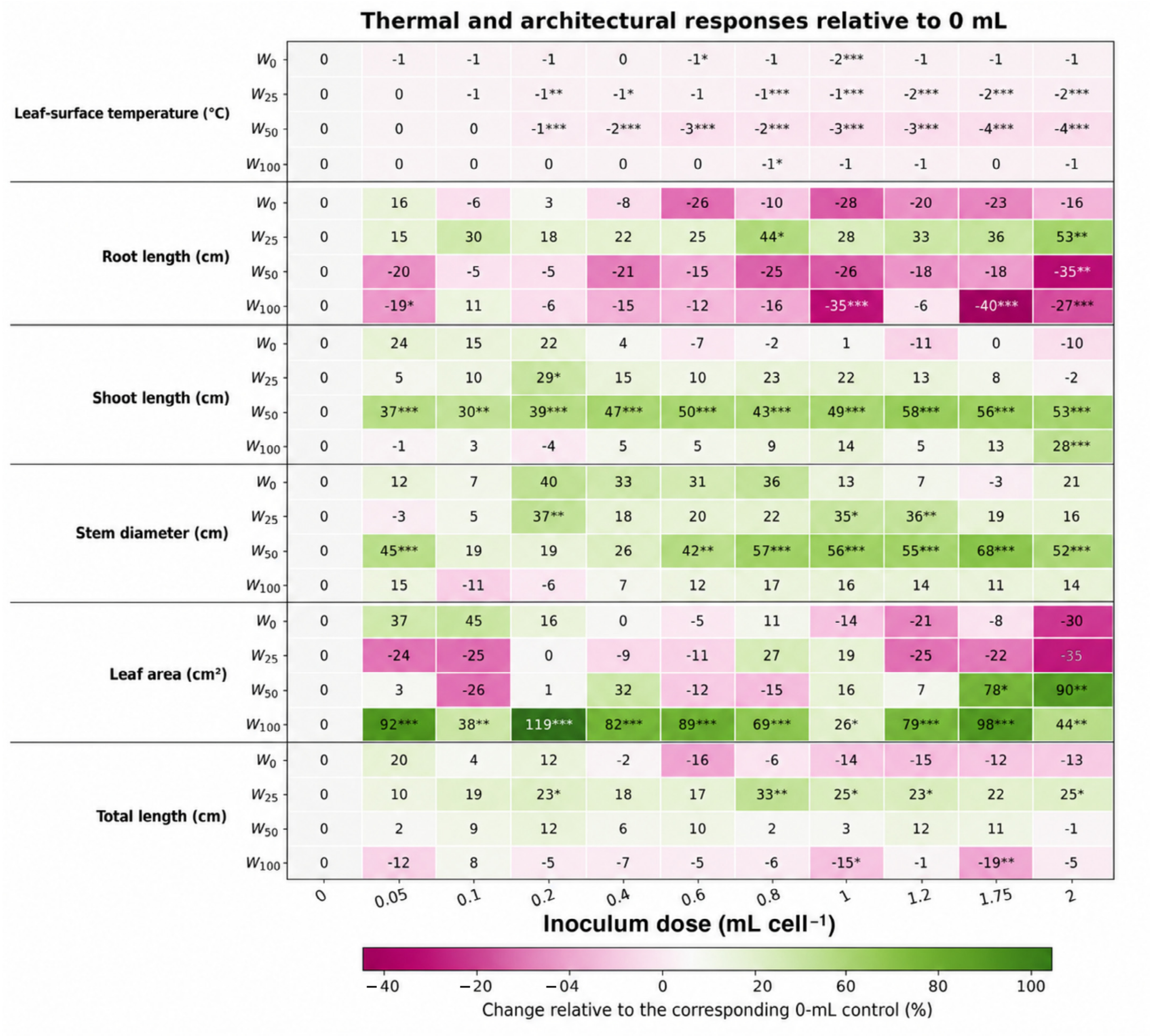

Figure S2. Percentage changes in the leaf-surface temperature and architectural traits relative to those of the corresponding 0-mL controls within each water regime. Values were calculated from the treatment-cell means; positive values indicate increases, and negative values indicate decreases. Asterisks indicate the significance of the Holm-adjusted model-based contrast against 0 mL within the same water regime: *, adjusted $p < 0.05$; **, adjusted $p < 0.01$; ***, adjusted $p < 0.001$. Each mean represents six independent tray cells.

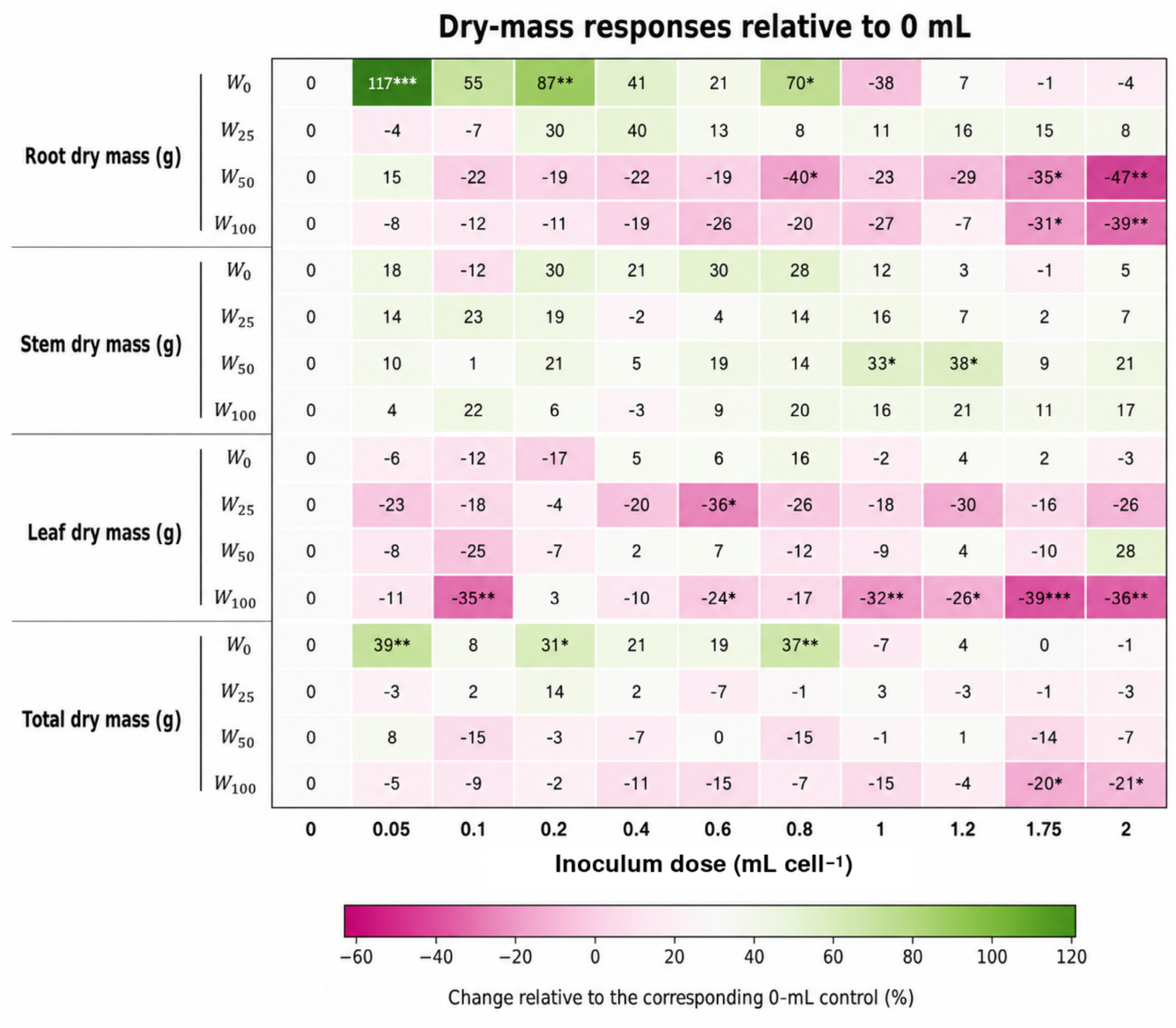

Figure S3. Percentage changes in root, stem, leaf and total dry mass relative to those of the corresponding 0-mL control for each water regime. Positive values indicate increases, and negative values indicate decreases. Asterisks indicate Holm-adjusted dose-versus-control contrasts: *, adjusted $p < 0.05$; **, adjusted $p < 0.01$; ***, adjusted $p < 0.001$. Each treatment-cell mean was calculated from six independent tray cells.

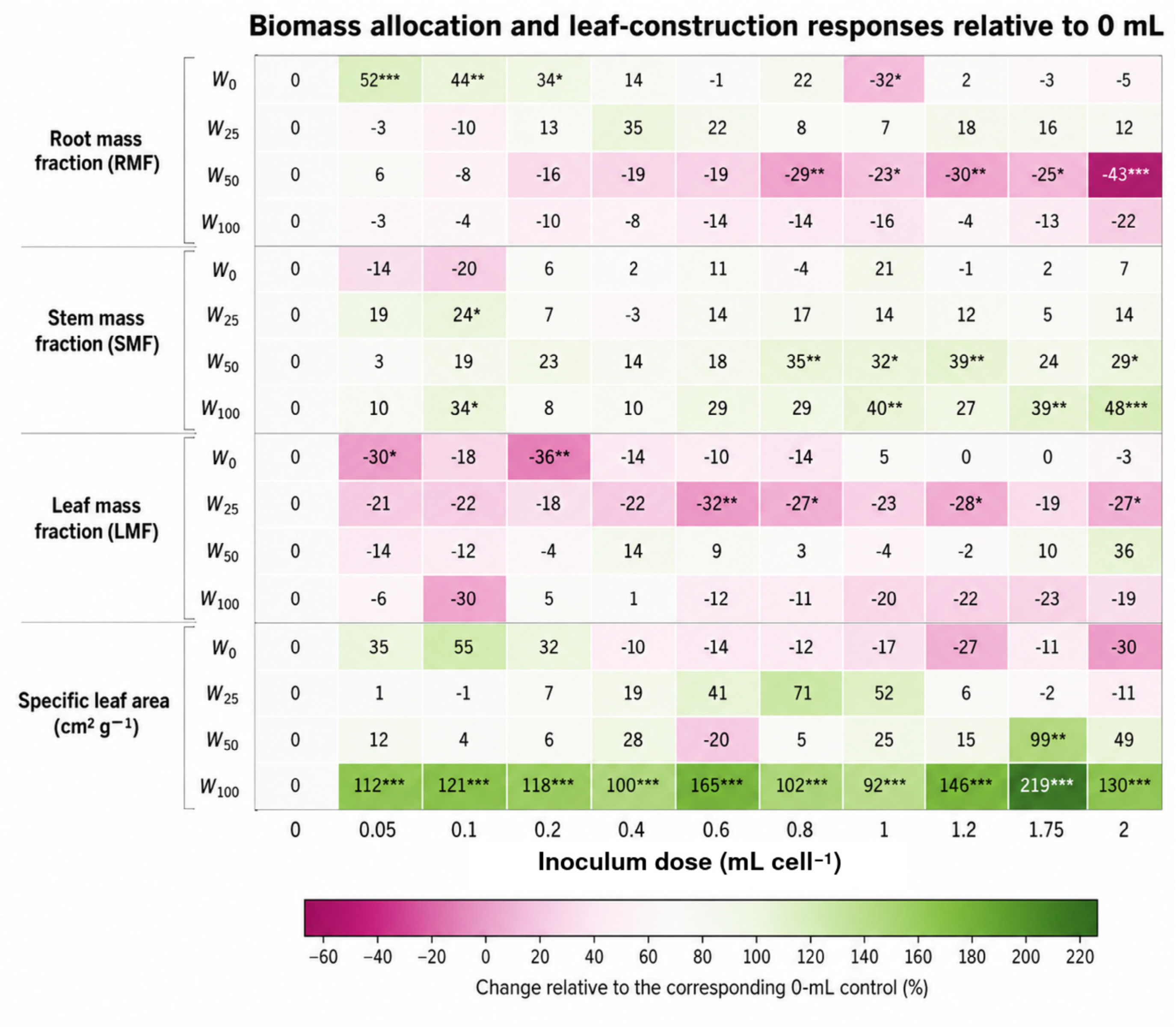

Figure S4. Percentage changes in the root mass fraction (RMF), stem mass fraction (SMF), leaf mass fraction (LMF) and specific leaf area (SLA) relative to those of the corresponding 0-mL controls within each water regime. Positive and negative values denote increases and decreases, respectively. Asterisks indicate Holm-adjusted dose-versus-control contrasts: *, adjusted $p < 0.05$; **, adjusted $p < 0.01$; ***, adjusted $p < 0.001$. Each treatment-cell mean was calculated from six independent tray cells. The mass fraction values are expressed as g $g^{-1,}$ and the SLA is expressed as cm^2 g^{-1}.

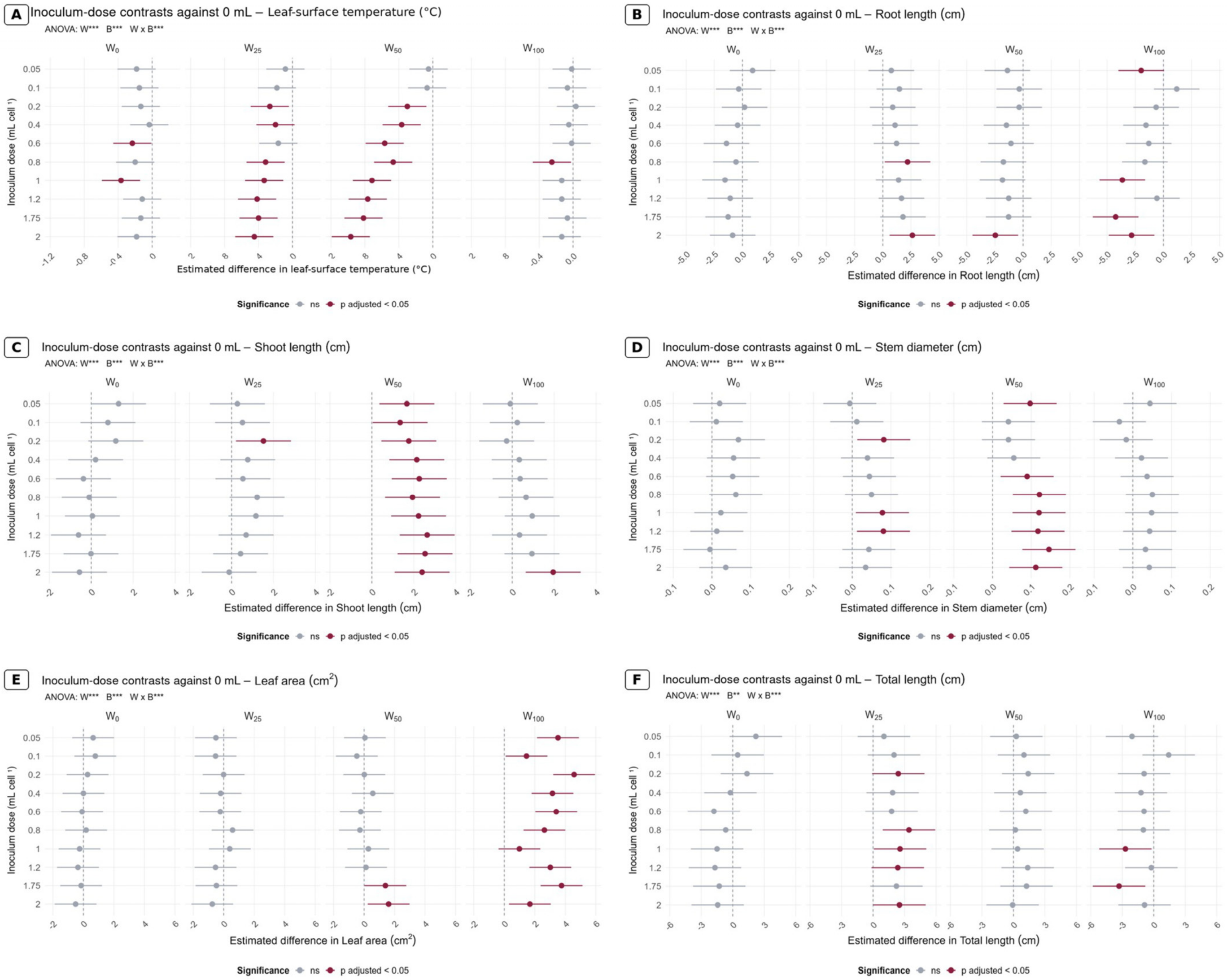

Figure S5. Model-based inoculum-dose contrasts against the corresponding 0-mL control for **(A)** leaf-surface temperature, **(B)** root length, **(C)** shoot length, **(D)** stem diameter, **(E)** leaf area and **(F)** total length. Points are estimated differences, and horizontal lines are 95% confidence intervals. Red symbols indicate Holm-adjusted $p < 0.05$; grey symbols are non-significant. The dashed vertical line denotes no difference. The compact statistics above each panel report the factorial ANOVA effects of W, B and W × B.

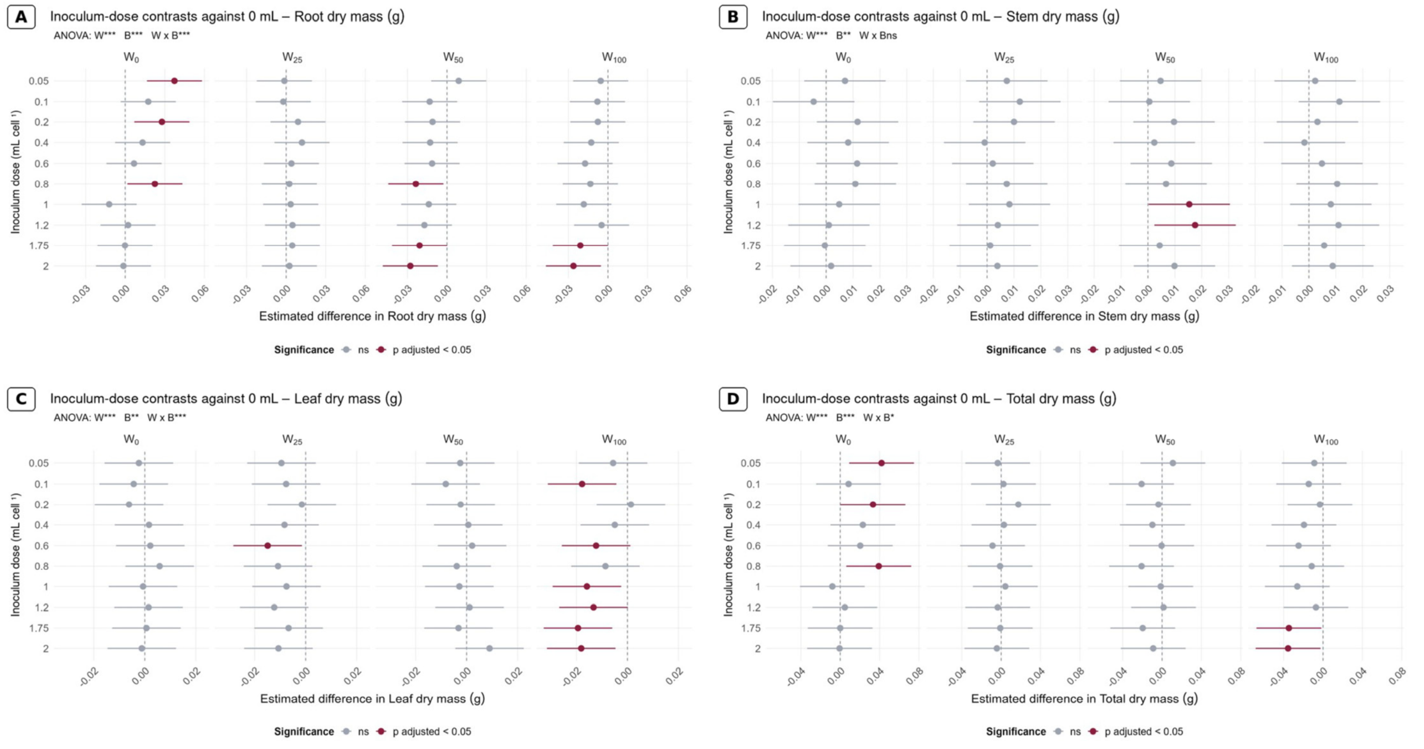

Figure S6. Model-based inoculum-dose contrasts against the corresponding 0-mL control for **(A)** root dry mass, **(B)** stem dry mass, **(C)** leaf dry mass and **(D)** total dry mass. Points are estimated differences, and horizontal lines are 95% confidence intervals. Red symbols indicate Holm-adjusted $p < 0.05$; grey symbols are non-significant. The dashed vertical line denotes no difference. The compact statistics above each panel report the factorial ANOVA effects of W, B and W × B. The estimated dry-mass differences are expressed as g plant^{-1}.

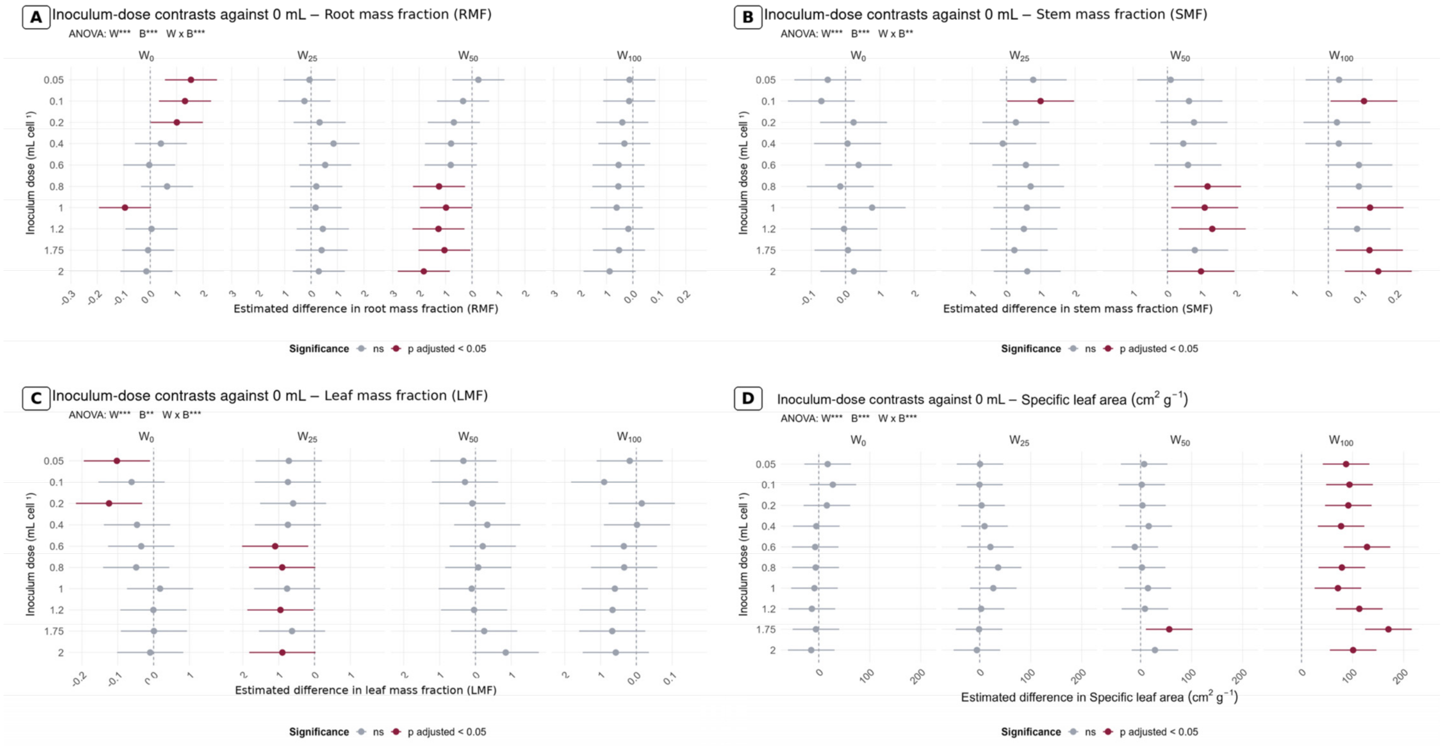

Figure S7. Model-based inoculum-dose contrasts against the corresponding 0-mL control for **(A)** root mass fraction (RMF), **(B)** stem mass fraction (SMF), **(C)** leaf mass fraction (LMF) and **(D)** specific leaf area (SLA).

Points are estimated differences, and horizontal lines are 95% confidence intervals. Red symbols indicate Holm-adjusted $p < 0.05$; grey symbols are non-significant. The dashed vertical line denotes no difference. The compact statistics above each panel report the factorial ANOVA effects of W, B and W × B. The estimated mass-fraction differences are expressed as g $g^{-1,}$ and the SLA differences are expressed as cm^2 g^{-1}.

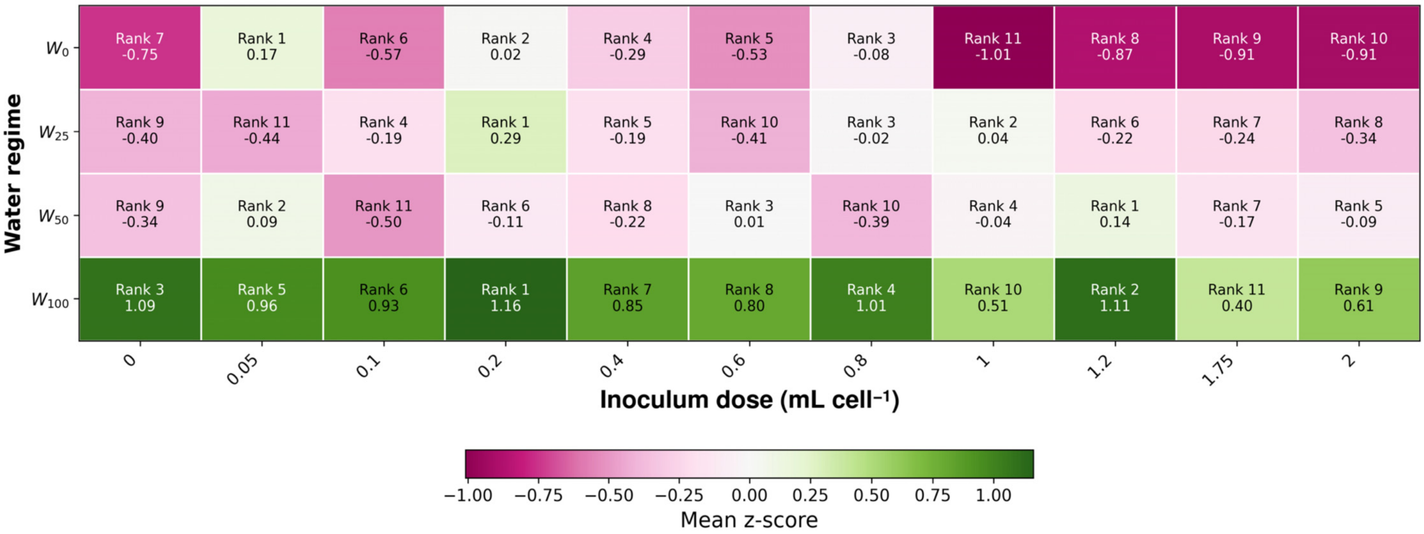

Figure S8. Integrated mean z-score and within-regime ranks for each water-regime × bacterial-dose combination. The score is the equal-weight arithmetic mean of the standardised treatment-cell means for leaf-surface temperature, root length, shoot length, stem diameter, leaf area, total length, root dry mass, stem dry mass, leaf dry mass and total dry mass. The leaf surface temperature was retained in its observed direction. Ranking was performed separately within W0, W25, W50 and W100.

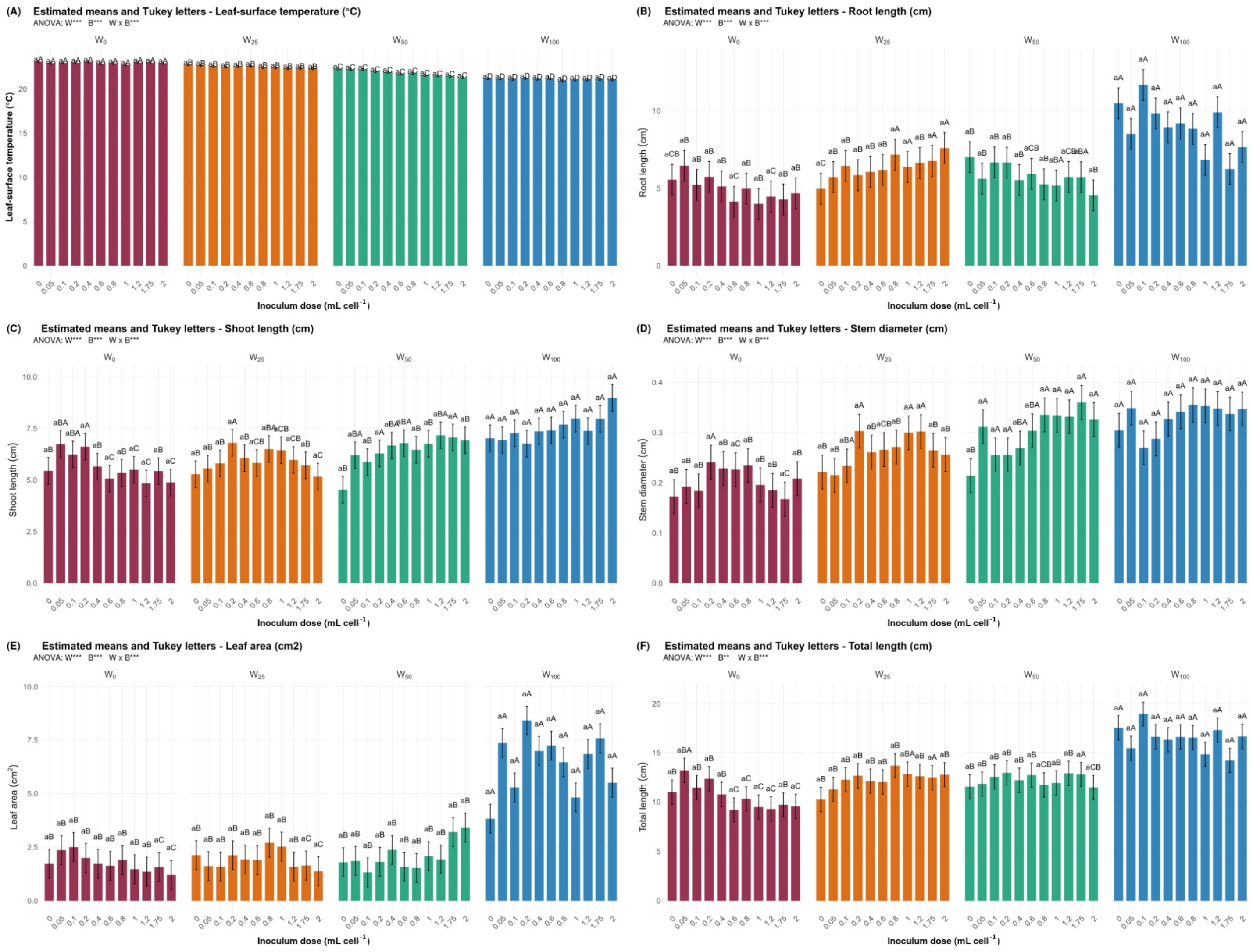

Figure S9. Estimated means and Tukey groupings for leaf-surface temperature and architectural traits: **(A)** leaf-surface temperature, **(B)** root length, **(C)** shoot length, **(D)** stem diameter, **(E)** leaf area and **(F)** total length. Bars show estimated marginal means, and error bars show standard errors for six independent tray-cell replicates per water regime x inoculum-dose combination. Lowercase letters compare inoculum doses within the same water regime, whereas uppercase letters compare water regimes within the same inoculum dose. Means sharing at least one letter do not differ, whereas means without a common letter differ in Tukey-adjusted all-pairwise comparisons at adjusted $p < 0.05$. Fixed-effect codes report the classical Type III two-way ANOVA for water regime (W), inoculum dose (B) and W × B.

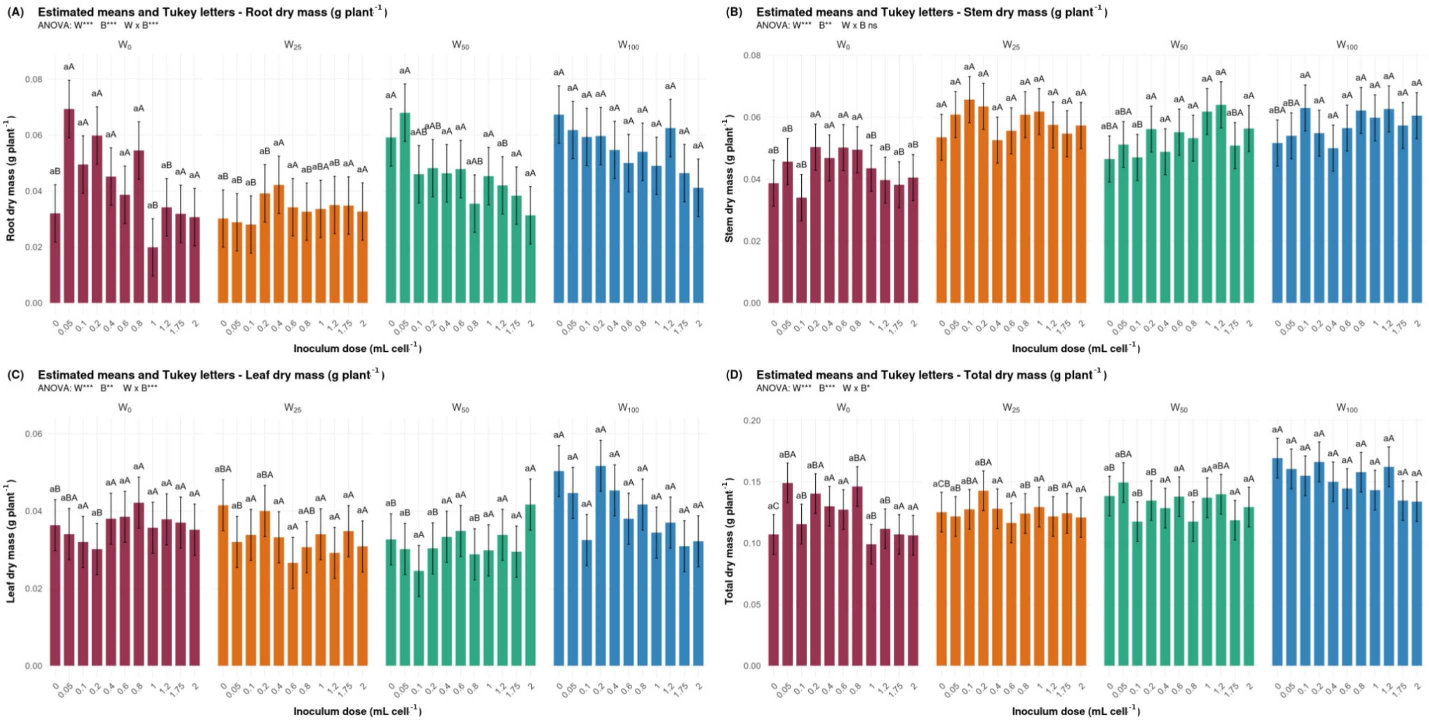

Figure S10. Estimated means and Tukey groupings for dry-mass traits: **(A)** root dry mass, **(B)** stem dry mass, **(C)** leaf dry mass and **(D)** total dry mass, expressed as g plant^{-1}. Bars show estimated marginal means, and error bars show standard errors for six independent tray-cell replicates per water regime x inoculum-dose combination. Lowercase letters compare inoculum doses within the same water regime, whereas uppercase letters compare water regimes within the same inoculum dose. Means sharing at least one letter do not differ, whereas means without a common letter differ in Tukey-adjusted all-pairwise comparisons at adjusted $p <$ 0.05. Fixed-effect codes report the classical Type III two-way ANOVA. For stem dry mass, W × B was non-significant in the classical analysis (p = 0.0552); the HC3 sensitivity analysis yielded p = 0.0147.

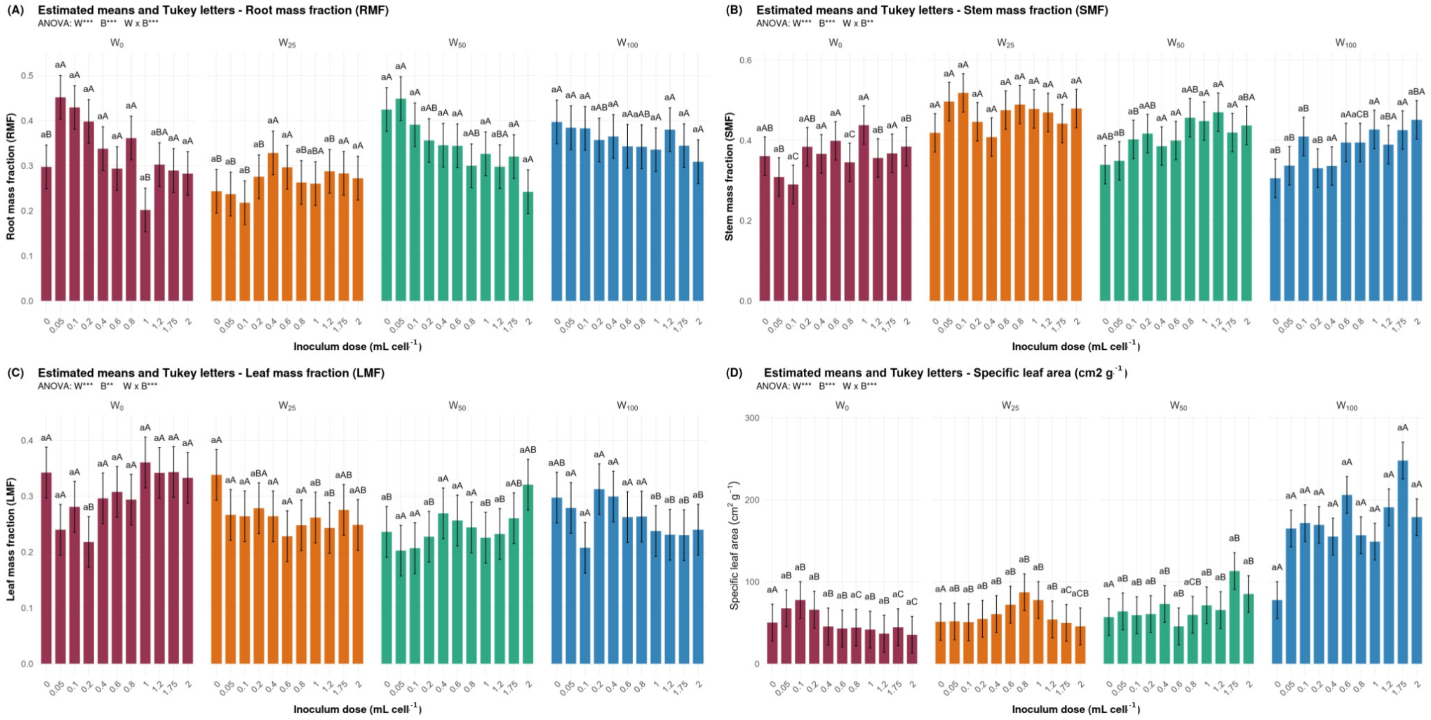

Figure S11. Estimated means and Tukey groupings for biomass allocation and leaf construction: **(A)** root mass fraction (RMF), **(B)** stem mass fraction (SMF), **(C)** leaf mass fraction (LMF) and **(D)** specific leaf area (SLA). Mass fractions are expressed as g g^{-1}, and SLA is expressed as cm^2 g^{-1}. Bars show estimated marginal means, and error bars show standard errors for six independent tray-cell replicates per water regime × inoculum-dose combination. Lowercase letters compare inoculum doses within the same water regime, whereas uppercase letters compare water regimes within the same inoculum dose. Means sharing at least one letter do not differ, whereas means without a common letter differ in Tukey-adjusted all-pairwise comparisons at adjusted $p <$ 0.05. Fixed-effect codes report the classical Type III two-way ANOVA.
